# Band Engineering and Structural-Geometrical Engineering in 2D/3D van der Waals Heterostructures for Advanced Photodetection and Intelligent Sensing

**DOI:** 10.1007/s40820-026-02129-4

**Published:** 2026-03-23

**Authors:** Miaomiao Yang, Kaiwen Gong, Yanxia Cui, Shaoding Liu, Guohui Li, Shenghuang Lin

**Affiliations:** 1 College of Physics and Optoelectronics, Key Laboratory of Interface Science and Engineering in Advanced Materials, Key Lab of Advanced Transducers and Intelligent Control System of Ministry of Education, Taiyuan University of Technology, Taiyuan, 030024 People’s Republic of China; 2 Songshan Lake Materials Laboratory Dongguan, Dongguan, 523808 Guangdong People’s Republic of China; 3 Dongguan Institute of Materials Science and Technology, Chinese Academy of Sciences, Dongguan, 523808 Guangdong People’s Republic of China

**Keywords:** 2D/3D heterostructures, Mixed-dimensional integration, Photodetectors, Optoelectronic applications

## Abstract

With the rapid advancement of the information era, the demand for device integration and intelligent sensing has grown significantly. Traditional three-dimensional (3D) materials are constrained by lattice mismatch and interfacial defects, and their limited functionalities often require bulky auxiliary components. In contrast, the rich family of two-dimensional (2D) materials eliminates lattice-matching constraints and offers unique light-matter interactions, paving the way for compact and novel intelligent sensing technologies. However, large-area fabrication and precise layer alignment in all-2D systems remain major challenges that hinder device scalability. Given that the performance and manufacturing capabilities of 2D materials cannot replace traditional semiconductors (such as Si), they are more likely to be heterogeneously integrated with conventional 3D semiconductors. 2D/3D heterojunctions combine the distinctive optoelectronic properties of 2D materials with the mature electronic functionalities of 3D semiconductors. In this work, we present recent advances in 2D/3D heterojunction photodetectors, with a particular emphasis on the underlying physical mechanisms, including band structure design, interface optimization, external-field coupling, and novel topological configurations. Meanwhile, we also explore emerging opportunities for CMOS-compatible and intelligent sensing optoelectronic systems. Finally, the challenges and future research directions toward the integrated development of 2D/3D heterojunctions are discussed.

## Highlights

- This review illustrates the application potential of 2D/3D systems from the perspectives of device fabrication, device performance, and system integration.
- This review examines implementation strategies for high-performance and intelligent 2D/3D heterojunction photodetectors, including band modulation, interface engineering, electric-field coupling, and geometric/structural design.
- This review provides a comprehensive discussion of the respective advantages and limitations of different modulation strategies.

## Graphical abstract

**Figure:**
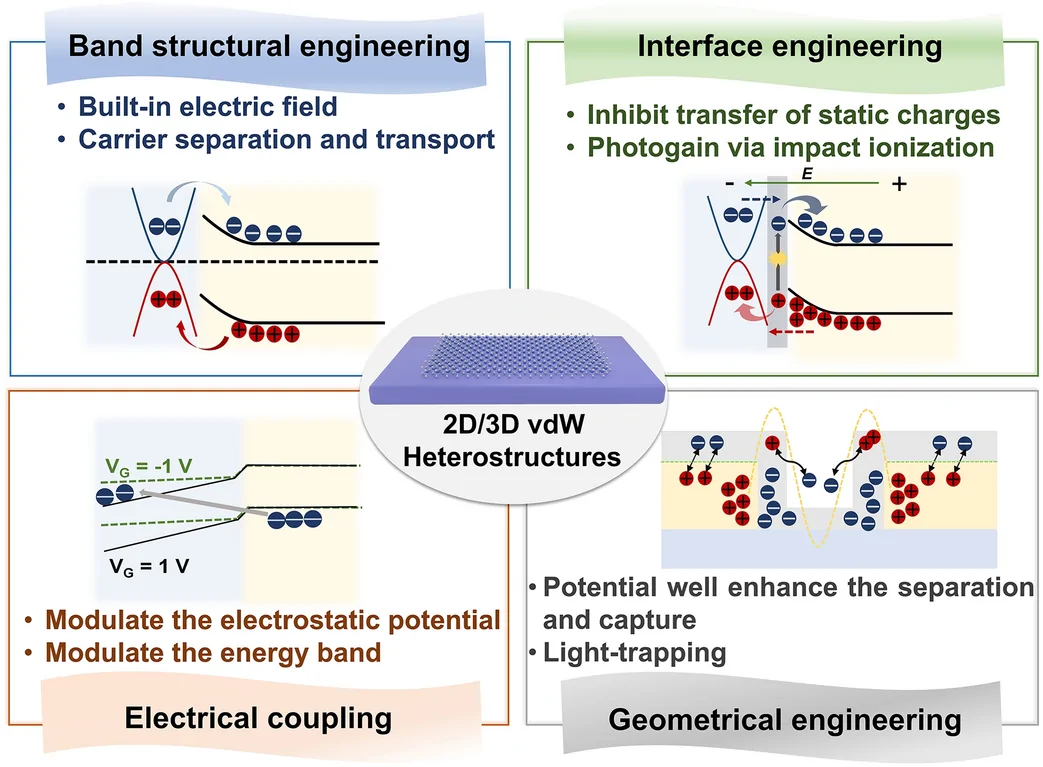

## Introduction

Two-dimensional (2D) materials have emerged as a transformative platform for optoelectronics, owing to their atomic thickness, quantum confinement, and van der Waals (vdW) interlayer coupling [1–3]. Unlike conventional 3D semiconductors, the weak vdW coupling in 2D materials endows them with many unique physical properties. For example, the dynamics of carriers and excitons are jointly influenced by interlayer coupling, quantum confinement, and environmental screening effects, resulting in highly distinctive interlayer energy transfer, exciton recombination, and migration behaviors, which in turn give rise to quantized electronic structures, tunable bandgaps, and highly accessible surface states [4–8]. In contrast, 3D materials are typically composed of bulk crystals or thin films, with layers bonded by strong covalent interactions. As a result, their electronic, excitonic, and carrier transport dynamics generally follow classical scattering and diffusion mechanisms, offering limited flexibility for achieving highly tunable interlayer coupling or pronounced quantum confinement effects (as shown in Table 1) [9]. It should be noted that the atomic-scale thickness of 2D materials imposes certain limitations in devices requiring strong light-matter interactions, such as photodetectors, nonlinear optical devices, or photovoltaic cells. Even though the intrinsic light-matter coupling per unit thickness is high, the extremely limited interaction length in 2D materials restricts the interaction time between photons and the active channel, resulting in weak optical absorption, particularly in the infrared range [10]. By comparison, 3D materials, with larger film or bulk geometries, allow for more flexible tuning and extension of the light-matter interaction length, making them inherently advantageous for devices requiring strong optical absorption or enhanced interaction strength [11].

**Table 1:** Performance comparison between 2D materials and 3D materials

Comparison	2D materials	3D materials
Size	~ Atomic thick	> 50 nm
Light-matter interaction	Interaction length is short; properties do not increase linearly with layer thickness	Enhancing light-matter interaction; performance can be extended with thickness
Interlayer coupling	Weak coupling enables unique phenomena such as interlayer exciton formation, ultrafast carrier transfer, interlayer energy transfer, and optical modulation	Strong interlayer coupling; Quantum control is relatively limited
Tunability and functional design	Easily tunable electronic, optical, and magnetic properties via stacking, strain, or interface engineering	Functional design mainly relies on intrinsic material properties; tuning methods are limited

Importantly, the absence of dangling bonds and lattice-matching constraints enables their integration with a broad spectrum of materials, ranging from conventional semiconductors to metals and dielectrics [12–15]. Artificially stacking 2D heterostructures via vdW interactions has been shown to preserve the intrinsic properties of 2D materials even in multilayer or bulk forms [16–21]. This is evidenced by phenomena such as the quantum Hall effect [22], excitonic condensation [23] and ultrafast carrier dynamics [24] observed in graphite and other multilayer 2D systems. 2D heterostructures offer an ideal platform for the efficient generation and transport of energetic carriers while maintaining the intrinsic crystal structure and inherent physical properties of the constituent materials. Such unique characteristics not only enable the advancement of fully 2D devices but also support the synergistic integration of 2D and 3D systems, thereby surpassing the performance and functional limitations of conventional devices [25, 26].

**Fig. 1:**
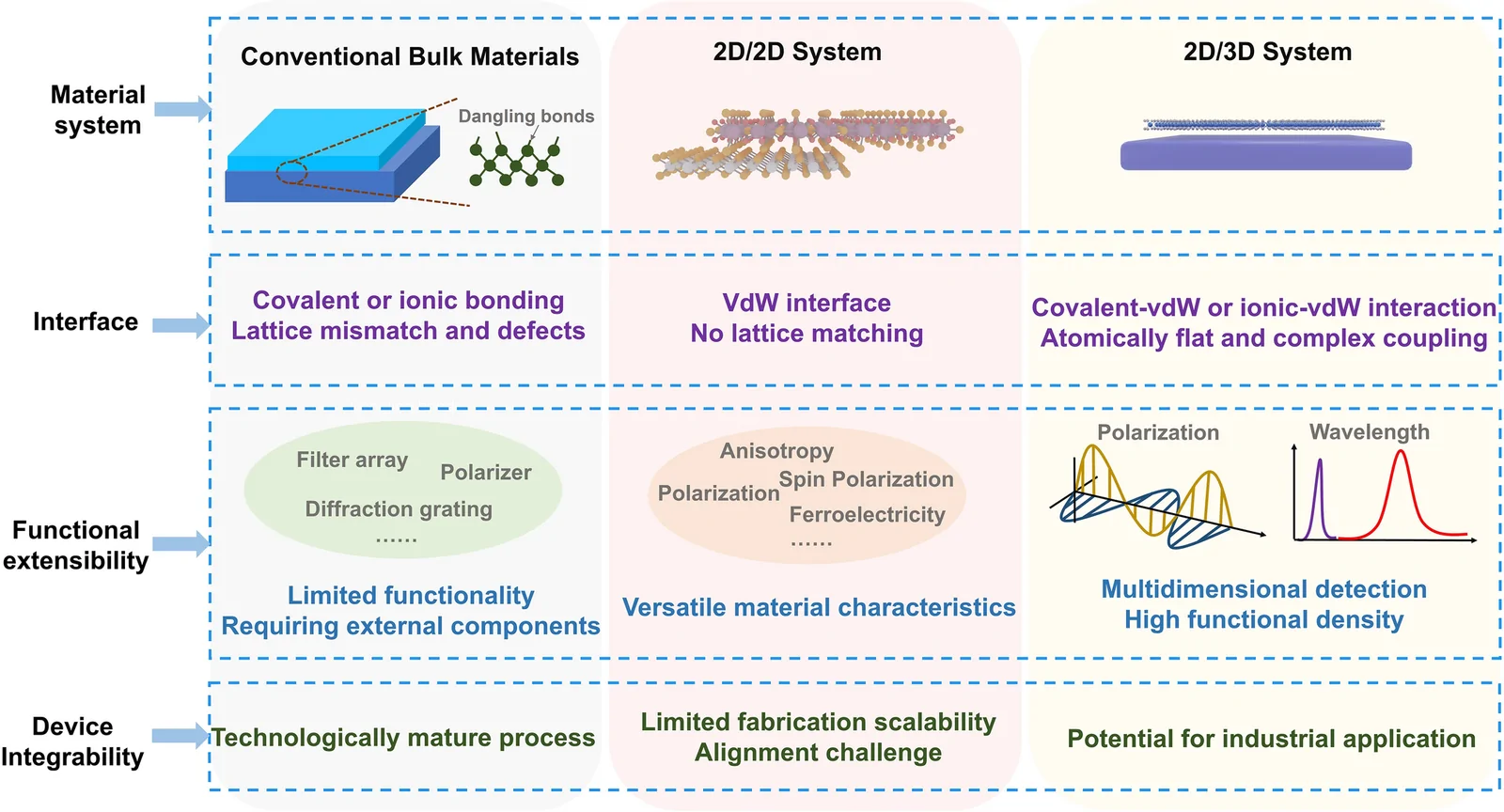
Comparative study of 2D/3D system versus bulk materials and 2D/2D system in terms of functionality and integration

Fully 2D systems have enabled a wide range of multifunctional photodetectors, including broadband photodetection [25, 27], polarization-sensitive detection [28], wavelength-selective response [29], and low-power neuromorphic sensing [30, 31]. However, due to intrinsic limitations in precise interlayer alignment and large-area scalability, the mass production of fully 2D systems remains challenging and currently falls short of meeting the consistency and reliability requirements for industrial applications. Compared with conventional semiconductor platforms and fully 2D systems, 2D/3D heterojunctions, defined as hybrid structures formed by the vertical integration of atomically thin 2D materials with bulk 3D materials, uniquely combine the atomically thin geometry, ultraclean vdW interfaces, and highly tunable electronic structures of 2D materials with the mature processing infrastructure and superior mechanical and optoelectronic performance of bulk semiconductors, demonstrating clear potential for scalable manufacturing and broad technological applications. Notably, owing to the extreme thinness and interfacial sensitivity of the 2D layers, these 2D/3D systems exhibit a rich spectrum of interfacial coupling phenomena (Fig. 1) [32]. In 2D/3D heterostructures, such interfacial coupling enables multifunctional capabilities, including ultrafast carrier transfer [33] and Schottky barrier modulation in the electrical domain [34], tunable optical modulation and enhanced absorption in the optical domain [35], and reduced device junction temperature and improved reliability through the high thermal conductivity of 2D materials in the thermal domain [36]. Beyond these interfacial effects, the exceptional optoelectronic response of 2D materials renders them ideal functional layers for multidimensional sensing and low-power signal processing. The integration of 2D and 3D materials thus provides a feasible pathway toward substantially enhanced functional density and information-processing efficiency [37, 38].

In recent years, significant progress has been made in 2D/3D heterojunction photodetectors, and several review articles have partially discussed the overall development trends in this field [11, 39–42]. However, existing literature predominantly focuses on the role of 3D material selection in modulating the heterojunction band structure and its impact on device performance, or emphasizes the application potential of 2D/3D heterojunctions in broadband photodetection. Nevertheless, these studies often lack in-depth analysis of the underlying physical mechanisms driving performance enhancement and fail to systematically summarize feasible optimization pathways for achieving high-performance devices. In fact, realizing substantial breakthroughs in overall device performance requires not only an understanding of the intrinsic material properties but also a comprehensive exploration of key factors influencing performance from multiple dimensions, including band structure engineering, interface state control, and structural design. Such insights are essential for guiding the future design and fabrication of advanced photodetectors.

In this review, we systematically analyze the application advantages of 2D/3D heterojunctions and provide a comprehensive summary of the current research progress in 2D/3D heterostructures, including both conventional and functional 3D semiconductor nanomembranes. From a mechanistic perspective, we summarize the latest advances in 2D/3D heterojunction photodetectors from several key viewpoints, including band structure design, interface optimization, external-field coupling, and geometric/structural engineering. We further provide a detailed analysis and discussion of the main focuses and synergistic effects associated with different modulation strategies. In addition, we review emerging applications in imaging, intelligent machine vision, logic operations, and integrated optoelectronic systems, highlighting the synergistic interplay between 2D materials and established semiconductor platforms. Finally, we discuss the current challenges and prospective opportunities regarding device performance, structural design, fabrication, and system-level integration, providing guidance for the development of next-generation, multifunctional, and CMOS-compatible optoelectronic technologies based on 2D/3D van der Waals heterostructures.

## Application Potential of 2D/3D Heterojunctions

The single-component photodetectors often suffer from inherent trade-offs among response speed, responsivity, and detectivity [43]. In contrast, vdW heterostructure-based devices can substantially alleviate these constraints through band engineering, interface optimization, and multi-physical-field coupling, enabling the simultaneous enhancement of multiple performance metrics [44–47]. While vdW hetero-integration has been extensively explored across diverse material systems, an increasingly central question is whether this approach possesses genuine technological feasibility and industrial potential beyond laboratory demonstrations.

Some studies suggest that large-area vdW integration of 2D materials with 3D semiconductors and lithographically defined 3D micro-/nanostructures can significantly reshape this landscape [48, 49]. This strategy preserves the intrinsic merits of vdW integration such as atomically clean interfaces, sharp electronic properties, and low interfacial defect densities while leveraging the superior scalability and process maturity of 3D semiconductor materials [11]. As a result, it offers a realistic pathway toward high-uniformity device arrays and system-level integration. Compared with fully 2D vdW heterostructures and conventional 3D devices, 2D/3D integration exhibits several distinctive and critical advantages across multiple dimensions (Fig. 1).

From the perspective of device fabrication and manufacturability, although the physical assembly of individual 2D building blocks into 2D/2D vdW heterostructures provides exceptional flexibility for heterogeneous integration and has greatly accelerated fundamental studies and proof-of-concept demonstrations, achieving high yield, high throughput, and high uniformity across large-scale device arrays remains highly challenging [50–52]. This limitation primarily arises from the immaturity of large-area, highly uniform 2D material growth techniques, and in 2D/2D heterostructures, the sequential alignment and transfer of multiple 2D layers further amplify these uncertainties. In contrast, 3D materials can be fabricated with large area and excellent uniformity using well-established processes such as epitaxial growth, thin-film deposition, and micro-/nanofabrication, thereby significantly reducing reliance on precise interlayer alignment [53]. Consequently, 2D/3D integration offers superior manufacturability and process robustness, and can be regarded as an effective transitional strategy to bridge the gap between frontier 2D materials research and industrial-scale applications.

In terms of device performance and functional expansion, the exceptional electrical and optical properties of 2D materials can synergistically complement the mature device architectures and robust bulk properties of conventional 3D semiconductors, introducing a higher-dimensional design space for device regulation [2]. By incorporating 2D functional layers onto 3D semiconductor platforms, novel physical effects and functionalities that are difficult to realize in traditional device systems can be achieved without substantially increasing structural complexity. Representative examples include polarization photodetection [54], reconfigurable photoresponse [55], and neuromorphic sensing [56], all of which play a critical role in enhancing the functional density and versatility of individual devices.

From the perspective of system integration and practical deployment, 2D/3D integration exhibits clear advantages over fully 2D heterostructures in terms of process compatibility, enabling more seamless co-integration with mainstream semiconductor manufacturing technologies such as CMOS processes [53]. The rapid development of 3D semiconductor nanomembranes and their transfer techniques has further facilitated the emergence of vdW-enabled layer-by-layer stacking architectures [57, 58]. Through the sequential stacking of pre-fabricated active or passive device layers including semiconductor channels, gate dielectrics, electrodes, CMOS circuits, memory units, and photodiode arrays highly integrated heterogeneous stacking systems can be constructed [59, 60]. Individual functional layers may be separated by planarization layers and interconnected through vertical vias, allowing efficient system-level coupling while preserving device independence. Such heterogeneous stacking architectures are free from stringent lattice-matching and process-compatibility constraints, significantly reducing integration complexity and manufacturing cost while enabling compact footprints and high integration density. Moreover, 3D semiconductor platforms already possess mature data-processing infrastructures and well-established process ecosystems, including highly reliable CMOS circuitry and memory technologies. Within this framework, 2D/3D integration allows 2D materials to serve as high-performance, low-power front-end sensing or modulation layers that can be efficiently coupled with back-end mature 3D electronic and photonic systems.

## Modulation Strategies for 2D/3D Heterojunctions

**Fig. 2:**
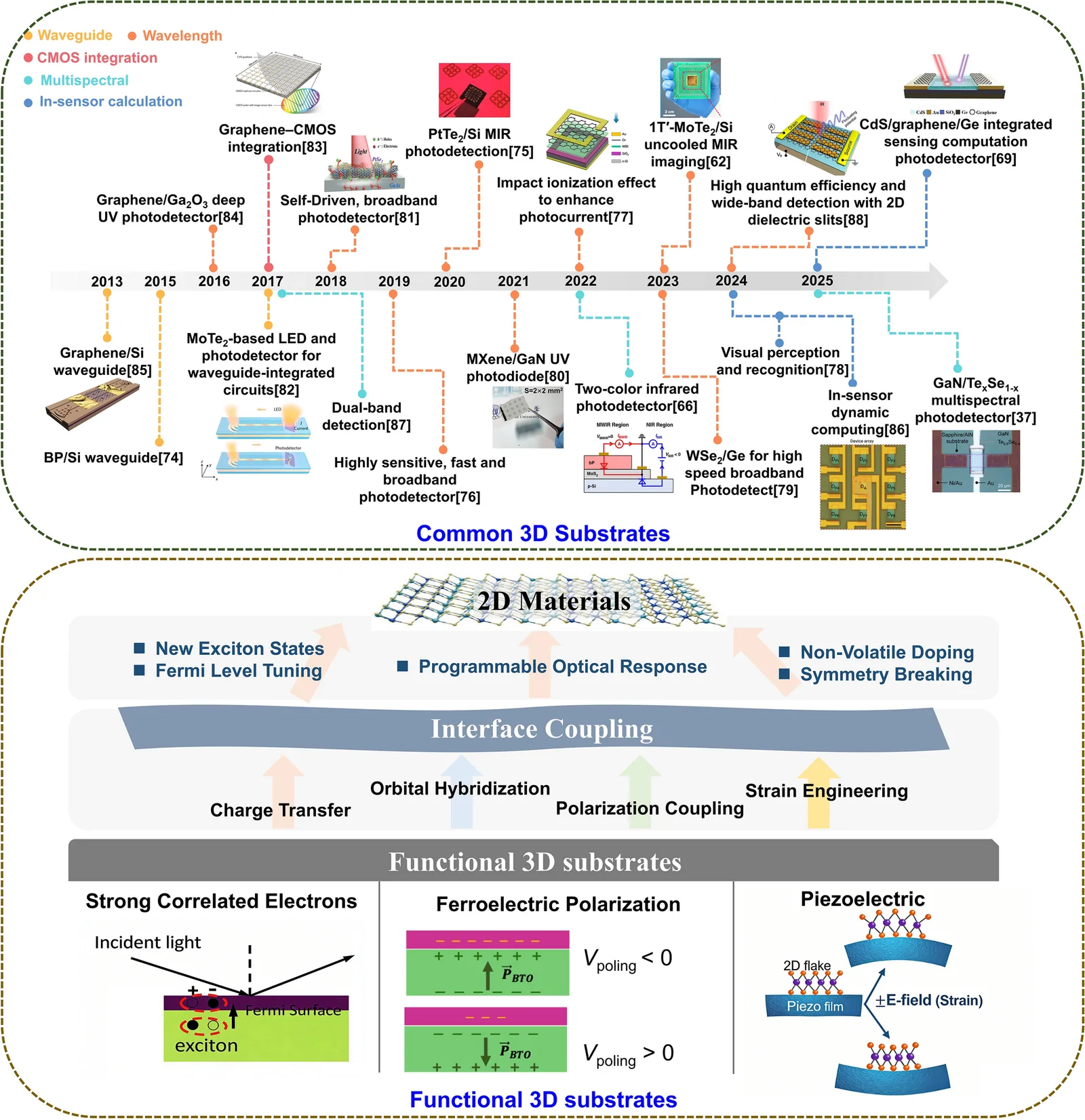
2D/3D Heterostructures: timeline, interface coupling, and functional modulation. Reprinted with permission from Refs. [37, 62, 66, 69, 74–88]

Early studies on 2D/3D photodetectors primarily focused on enhancing wavelength detectability, including the development of broadband and multiband photodetectors (as shown in Fig. 2). These efforts leveraged the rich and continuously tunable material library of 2D materials [61]. For example, devices based on 2D/3D heterojunctions have already achieved room-temperature infrared detector arrays operating at 10.6 µm [62]. However, as application scenarios become increasingly complex, operating environments more variable, and performance requirements continue to rise, traditional photodetectors with fixed spectral response ranges have gradually revealed their limitations. Under complex backgrounds and strong noise conditions, such devices often struggle to effectively distinguish targets from the background, exhibiting insufficient interference immunity, high false-alarm rates, and poor adaptability to environmental factors such as illumination variations and temperature fluctuations. These issues severely constrain their further deployment in applications requiring high reliability and high precision. To overcome these bottlenecks and enhance the overall sensing capability of detection systems, multispectral detection technologies have attracted growing attention and have developed rapidly [63, 64]. By simultaneously acquiring the optical responses of targets across multiple spectral bands, multispectral detection not only improves interference resistance but also reveals intrinsic spectral characteristics of targets at different wavelengths, thereby providing richer and more discriminative information dimensions. Through rational design of heterojunction band alignment, photodetectors operating at dual-band [37, 65, 66] or even triple-band wavelengths [67] have become feasible.

To further enable miniaturization and promote chip-level integration, recent advances have driven the development of on-chip polarization-sensitive photodetectors and in-sensor computing architectures. In particularly, compared with polarization-sensitive photodetectors based on naturally or artificially anisotropic absorbing materials [68] which are typically limited to linear or circular polarization detection structural designs based on 3D materials have enabled full-Stokes detection [48]. Moreover, by combining heterojunction structural design with external-field coupling to exploit optically modulated threshold controllability [69], tunable response directionality [70], and wavelength-selective photoresponse [71], brain-inspired computing functionalities have been realized. These advanced sensors are capable of performing visual computing tasks directly at the sensing front end, including noise suppression, convolution operations, feature extraction, object recognition, and pattern classification, thereby reducing data transmission requirements and energy consumption [56, 72, 73].

With the continuous maturation of epitaxial growth and layer lift-off techniques, 3D materials have achieved remarkable progress in thin-film realization, giving rise to a class of freestanding nanomembranes featuring vdW interfaces [53, 89]. A wide range of conventional bulk photonic materials have been successfully exfoliated into single-crystalline nanomembranes, which preserve their intrinsic optical, electronic, and nonlinear properties while offering precisely controllable thickness, transferability, and integrability [90]. For example, freestanding Si films have been employed in flexible optoelectronic systems [91], III-V semiconductor nanomembranes (such as GaAs [92] and InP [57]) have enabled high-performance heterogeneously integrated lasers, photodetectors, and optical amplifiers on silicon photonic platforms, and wide-bandgap III-nitride nanomembranes (such as AlN [93] and GaN [58]) have demonstrated unique advantages in electro-optic modulation and piezoelectric photonic devices. Unlike 2D materials, the optical and electronic properties of 3D freestanding nanomembranes are generally insensitive to thickness variation, allowing for substantially enhanced light-matter interaction lengths and making them more suitable for applications such as optical amplification, modulation, and frequency conversion [90]. More importantly, these nanomembranes can be transferred and coupled to arbitrary substrates or photonic architectures via vdW integration, thereby circumventing lattice-matching constraints and the limitations of conventional epitaxial approaches, and offering new material and structural degrees of freedom for high-performance heterogeneously integrated photonic and optoelectronic devices [94]. Beyond conventional bulk materials, functional oxide crystals in the 3D form, such as BaTiO_3_ [95], LiNbO_3_ [96] and SrTiO_3_ [97] have also been realized as high-quality freestanding thin films and heterogeneously integrated. Beyond extending the spectral response through material selection and band engineering of 2D materials, unlike conventional substrates (such as Si), the intrinsic functional properties of 3D substrates also play a crucial modulatory role in 2D/3D heterostructures, as illustrated in the lower half of Fig. 2.

Functional 3D substrates can introduce additional order parameters and coupling channels into adjacent 2D materials, thereby enabling synergistic performance enhancements beyond what can be achieved with 2D material systems alone. Functional 3D substrates (e.g., ferroelectric, piezoelectric, or correlated oxides) enable active modulation of the electronic structure, optical response, as well as excitonic and valley degrees of freedom of adjacent 2D materials through interfacial multiphysics coupling mediated by their intrinsic order parameters [98].

At the most fundamental level, ferroelectric 3D substrates with strongly correlated electronic states can reshape the carrier behavior and band structure in 2D materials through interfacial charge transfer and doping. Charge redistribution driven by work-function mismatch, polar surface terminations, or interfacial chemical bonding can effectively tune the Fermi level position, conduction type, and carrier concentration of the 2D material, thereby influencing its photoconductivity and excitonic state populations [99]. Beyond simple electrostatic effects, atomic-scale orbital hybridization can modify many-body interactions and electronic correlation strength in the 2D material. As demonstrated by Yin et al., when monolayer MoS_2_ is integrated with the strongly correlated electronic system SrTiO_3_, high-energy excitonic wavefunctions can propagate from the substrate into the 2D material. More importantly, such strong spin–orbit-charge-lattice coupling may induce the emergence and splitting of new high-energy excitonic resonances and even generate Fermi-surface features at the interface, leading to pronounced modulation of the intrinsic optical response and electronic density of states in the 2D material [100].

From the perspective of symmetry, 3D substrates with ferroelectric or piezoelectric functionalities can introduce nonvolatile polarization fields or strain fields at the interface, thereby breaking the intrinsic spatial inversion symmetry of 2D materials [98, 101, 102]. This symmetry breaking not only induces band Stark effects and lifts valley degeneracy, but also enables systematic control over the nonlinear optical responses of 2D materials, such as the intensity, polarization state, and phase of SHG. The study by Li et al. demonstrated that the in-plane polarization at ferroelectric domain walls of a PbZr_0.2_Ti_0.8_O_3_ (PZT) substrate interacts with the intrinsic polarization axis of MoS₂, allowing further control over the symmetry of SHG [103]. Patterning of ferroelectric domains offers the capability to program nonlinear optical polarization.

**Fig. 3:**
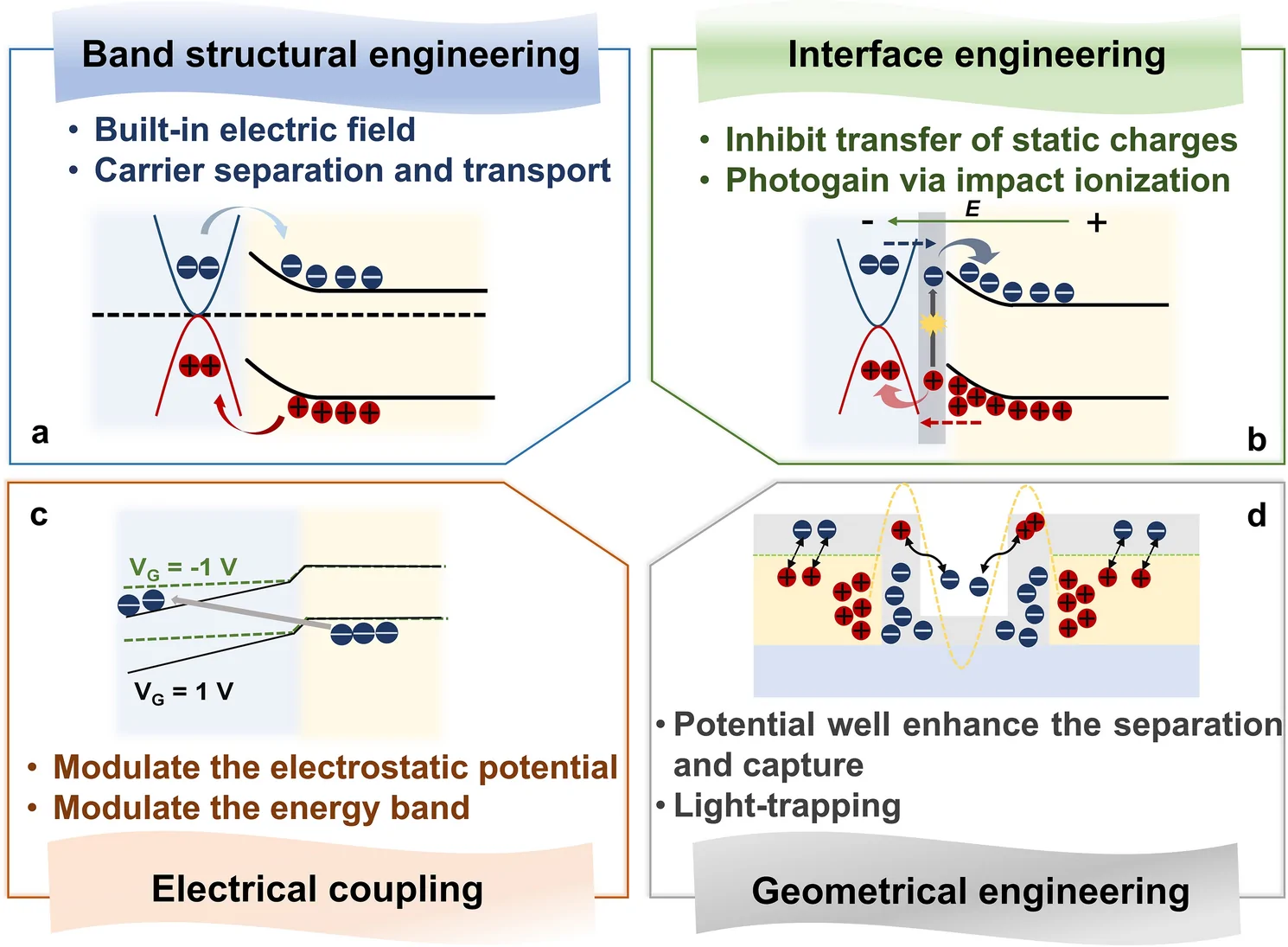
Key modulation strategies for 2D/3D heterojunction devices. **a** Band bending occurs at the interface, generating a built-in electric field, which enables the rapid separation of photogenerated charge carriers. **b** Under a high electric field, carriers can undergo quantum tunneling (green and red arrows), followed by impact ionization (black arrows), resulting in photocurrent multiplication. **c** Gate voltage can efficiently modulate the Fermi level and energy band positions of 2D materials (the arrows indicate the band-to-band tunneling characteristics). **d** Slit structure formed by the structural design transforms the enhanced built-in electric fields on both sides of the grating into a valley-shaped potential distribution, thereby enabling efficient separation of photogenerated carriers. The green dashed lines represent the interfacial electric field of the heterojunction, the yellow dashed lines indicate the slit-induced enhanced electric field, and the black arrows denote the combined effect of both fields on the separation of photogenerated carriers

With the advancement of 2D/3D heterojunction photodetectors, their applications have evolved from purely high-performance visual detection to multifunctional optoelectronic responses. As device functionalities diversify, understanding the underlying physical mechanisms becomes increasingly critical. In 2D/3D heterojunctions, performance can be modulated through band structure engineering, interface engineering, electrical coupling, and topological optimization, as shown in Fig. 3. Band structure engineering is pivotal for enhancing device performance by governing carrier generation, separation, and transport dynamics while simultaneously shaping junction energy barriers, interfacial recombination, and spectral response [104, 105]. Rational design of the 2D/3D band alignment thus enables efficient carrier injection and transport, enhancing response speed, sensitivity, and overall device performance. Beyond rational band structure design, precise control of the heterostructure interface is equally critical. Interface engineering not only enables the modulation of interlayer interactions but also effectively regulates the carrier transfer dynamics at the interface [106]. By introducing interface passivation layers, functional modifications, or molecular-layer engineering, interface engineering can further suppress dark current, enhance photodetection speed and sensitivity, and improve the long-term stability and reliability of the device [107, 108]. The deliberate design of interface layers can introduce additional photoelectric effects, such as carrier multiplication, enabling high gain or a broad dynamic response, and allowing the device to maintain excellent performance even under low-light conditions [77].

Electrical coupling, by modulating interfacial charge transfer and barrier distribution, enables multiple functional enhancements, including the control of doping types [68], the triggering of phase transitions [73] and the modulation of the Fermi level through carrier transfer across interfaces [55, 109]. Under electric-field control, device performance is no longer fixed but can be electrically reconfigured as needed, offering a promising approach for adaptive optoelectronics and neuromorphic visual chips, and providing an effective strategy for the fabrication of intelligent photodetectors [70]. The design of topological architectures further introduces reconfigurability in the structural dimension. The design of 3D topological architectures enables the application of highly directional and spatially graded mechanical strain to the 2D materials integrated on them by precisely engineering structural deformations, thereby achieving localized strain modulation at the nanoscale [110, 111]. Such mechanically induced fields can effectively tailor the band structure, carrier transport behavior, and local density of states of 2D materials, and can further generate position-dependent photocarrier distributions under illumination, leading to dynamic coupling between localized optical responses and global optoelectronic outputs. Consequently, the optoelectronic characteristics of the device can be reconfigured in real time, including spectral absorption shifts, enhanced responsivity, and controlled carrier migration pathways [88, 112].

## Advanced Key Performance Metrics of Photodetectors

With the rapid development of emerging semiconductor materials such as low-dimensional semiconductors, photodetectors based on these materials have demonstrated great potential for next-generation optoelectronic applications. However, due to the unique material properties, device architectures, and physical mechanisms involved (e.g., ion migration, charge trapping, and interfacial effects), the performance characterization of photodetectors based on emerging semiconductors faces several challenges. This section focuses on introducing advanced performance metrics for the evaluation of state-of-the-art photodetectors [113].

### Responsivity (R)

The responsivity characterizes a photodetector’s ability to convert incident optical signals into electrical signals and is defined as the photocurrent (or voltage) generated per unit incident optical power [106]. The calculation formula is:

1 $$R = \frac{{I_{ph} }}{{P_{{{\mathrm{in}}}} A}}$$

whereas ${I}_{ph}={I}_{\mathrm{light}}-{I}_{\mathrm{dark}}$ is defined as the photocurrent, ${P}_{\mathrm{in}}$ represents the incident optical power, and *A* denotes the illuminated area. The calculation should satisfy the following conditions: the device operates within the linear response regime over the measured optical power range; the incident light spot strictly matches the effective active area with a uniform illumination distribution; and the dark current remains stable during the measurement or has been subtracted in real time.

### External Quantum Efficiency

The external quantum efficiency (EQE) represents the ratio of the number of charge carriers collected by the device per unit time to the number of incident photons, serving as a quantized metric of photoelectric conversion efficiency [106, 114]. The calculation formula is:

2 $${\mathrm{EQE}} = \frac{{I_{ph} /q}}{{P_{{{\mathrm{in}}}} /\left( {h{\upnu }} \right)}} = \frac{Rhc}{{q\lambda }}$$

Here, ${I}_{ph}$ is the photocurrent, q is the elementary charge, ${P}_{\mathrm{in}}$ is the optical incident power, h is the Planck constant, ν is the photon frequency, c is the speed of light, and λ is the wavelength. In the calculation process, it is essential to ensure that all the considered photocurrent arises exclusively from effective photogenerated carriers. The EQE does not explicitly distinguish whether photoconductive gain is present in a device and therefore essentially represents an apparent quantum efficiency. In high-performance photodetectors, EQE values exceeding unity are frequently observed. This phenomenon primarily originates from photoconductive gain, whereby a single incident photon, once absorbed, can induce the generation and collection of two or more charge carriers. From a physical perspective, such gain arises from internal amplification processes within the device, including impact ionization under high electric fields, or more commonly, prolonged carrier lifetime induced by trap states and interfacial charge separation [114]. An extremely high EQE and photoconductive gain do not necessarily indicate superior optoelectronic performance. In devices without photoconductive gain, a high EQE can indeed directly reflect excellent photodetection capability. However, devices exhibiting large photoconductive gain often suffer from slow response speeds, potentially accompanied by persistent changes in conductivity. Therefore, the gain-bandwidth product is considered a more appropriate benchmarking metric for evaluating the overall performance of such photodetectors [113].

### Noise

Noise is a key factor determining the sensitivity, detectivity, and minimum detectable signal of photodetectors. The main types of noise in photodetectors include thermal noise, flicker noise, shot noise, generation-recombination noise, and photon noise. Thermal noise (Johnson-Nyquist noise) arises from the thermal motion of charge carriers in resistive elements and exists in all photodetectors with finite resistance. Flicker noise (1/f noise) is commonly observed in semiconductor devices and is mainly caused by the random capture and release of carriers in interface or bulk traps. Shot noise originates from the statistical fluctuations of carriers crossing junctions or electrodes. Photon noise arises from the quantum statistical fluctuations of incident photons. It is intrinsic to the photodetector and independent of device structure or readout circuitry, and therefore cannot be completely eliminated through device design. Thermal noise and shot noise have no characteristic time scale or memory effect. Their temporal fluctuations are completely random and mutually independent, so they can be regarded as ideal white noise.

In experiments and literature, the RMS value of the intrinsic noise current (${i}_{n,\mathrm{rms}})$ is commonly calculated using the noise power spectral density ${S}_{n}(f)$ and the equivalent noise bandwidth B:

3 $$i_{{n,{\mathrm{rms}}}} = \sqrt {S_{n} \left( f \right)B}$$

This formula is based on white noise (${S}_{n}(f)$ is frequency-independent). When the device noise is dominated by 1/*f* noise, this approximation is generally not applicable unless B≪f. Otherwise, significant errors may occur. Therefore, before using this formula, the frequency characteristics of the noise spectrum must be verified.

In cases where the noise power spectral density cannot be directly measured, researchers sometimes adopt the white-noise model to estimate the theoretical noise limit. In this case, the lower-limit noise values based on the white-noise model should be explicitly labeled as theoretical to distinguish them from experimental data. When theoretical, lower-limit noise values based on white-noise models are determined, choosing the appropriate model is crucial. For devices with photoconductive gain G, the theoretical shot noise can be expressed as (basic shot-noise model ${i}_{n,\mathrm{r}.\mathrm{m}.\mathrm{s}.,\mathrm{theor}.}=\sqrt{2q{I}_{\mathrm{dark}}B}$ is not applicable):

4 $$i_{{n,{\mathrm{rms}},{\mathrm{theor}}}} = \sqrt {2qI_{{{\mathrm{dark}}}} GFB}$$

where F is the Fano factor (with F=1 for independent carrier transport). To avoid underestimating the noise due to improper model selection, it is recommended to use a unified theoretical expression that accounts for both shot noise and thermal noise:

5 $$i_{{n,{\mathrm{rms}},{\mathrm{theor}}}} = \sqrt {2qI_{{{\mathrm{dark}}}} GFB + \frac{{4k_{B} TB}}{{R_{{{\mathrm{dark}}}} }}}$$

where the dark differential resistance is defined as $R_{{{\mathrm{dark}}}} = \frac{{{\mathrm{d}}V_{{{\mathrm{bias}}}} }}{{{\mathrm{d}}I_{{{\mathrm{dark}}}} }}$. It should be emphasized that this formula represents only the theoretical white-noise limit and cannot replace experimental noise measurements [113].

### Noise-Equivalent Power

Noise-equivalent power (NEP) is the most fundamental metric for evaluating the sensitivity of a photodetector. It is defined as the minimum incident optical power required to produce an output signal equal to the background noise under a specific bandwidth and frequency. A lower NEP indicates a stronger capability of the photodetector to detect weak optical signals. The basic definition is the incident optical power at which the signal-to-noise ratio (SNR) equals 1, under a specified bandwidth B and modulation frequency f.

To quantify the NEP and detectivity (*D*) of a photodetector, the device output power spectral density is typically measured while removing instrument noise. A modulated optical signal with root-mean-square power ${P}_{i,\mathrm{rms}}$ is applied, centered at frequency f and spanning bandwidth B. The ${P}_{i,\mathrm{rms}}$ at which the photodetector output equals the background noise power spectral density defines the NEP at that specific frequency and bandwidth, and its reciprocal gives the detectivity *D*. Alternatively, under suitable conditions, NEP can be calculated from the ratio of the root-mean-square noise current to the responsivity measured at an optical power approaching NEP, i.e., $\mathrm{NEP}={i}_{n,\mathrm{rms}}(f,B)/R(f,\lambda ,{P}_{\mathrm{rms}}=\mathrm{NEP})$. If the responsivity is measured only at optical powers far above NEP, or if the device exhibits nonlinear behavior near NEP, the resulting value often denoted as the apparent NEP serves only as an approximate reference and cannot replace direct experimental determination. Therefore, the most reliable method for accurately quantifying photodetector sensitivity remains the direct experimental measurement of the output power spectral density to extract NEP and *D*, while fully accounting for device noise characteristics, nonlinear response, and measurement conditions [113].

### Specific Detectivity

The detectivity D is defined as the reciprocal of the NEP and serves to characterize the sensitivity of a photodetector. The specific detectivity ${D}^{*}$ further eliminates the influence of the effective device area A and noise bandwidth B based on D, enabling performance comparison across different devices. Their calculation formulas are:

6 $$D = \frac{1}{{{\mathrm{NEP}}}}$$

7 $$D^{*} = \frac{{\sqrt {AB} }}{{{\mathrm{NEP}}}}$$

The fundamental assumption is that the noise current ${i}_{n,{\mathrm{rms}}}$ scales with area and bandwidth as ${i}_{n,{\mathrm{rms}}}\propto \sqrt{AB}$, and that both the device area and noise bandwidth are clearly defined. In practice, however, ${i}_{n,{\mathrm{rms}}}$ may exhibit non-$\sqrt{B}$ dependence or contain significant 1/f noise, and directly using ${D }^{*}$ may lead to misleading comparisons. To address this, a bandwidth-normalized specific detectivity can be introduced:

8 $$D_{{\overline{B}}}^{*} \left( {f,\lambda } \right) = \frac{{R\left( {f,\lambda ,P_{{i,{\mathrm{rms}}}} = {\mathrm{NEP}}} \right)\sqrt A }}{{i_{{n,{\mathrm{rms}}}} \left( {f,\overline{B}} \right)}}$$

where $R\left( {f,\lambda ,P_{{i,{\mathrm{rms}}}} = {\mathrm{NEP}}} \right)$ is the responsivity measured at modulation frequencyf, wavelength λ, and incident optical power equal to NEP, and $\overline{B}$ is typically taken as 1 Hz for convenient benchmarking. Theoretically, in the white-noise limit, the specific detectivity of a given device can be expressed as:

9 $$D_{{{\mathrm{theor}}}}^{*} \left( {f,\lambda } \right) = \frac{{R\left( {f,\lambda ,P_{{i,{\mathrm{rms}}}} = {\mathrm{NEP}}} \right)\sqrt A }}{{\sqrt {2qI_{{{\mathrm{dark}}}} GF + 4k_{B} T/R_{{{\mathrm{PD}}}} } }}$$

By jointly reporting $D_{{\overline{B}}}^{*}$ and $D_{{{\mathrm{theor}}}}^{*}$, one can achieve a reliable and comparable evaluation of photodetector performance while accounting for noise type and bandwidth dependence, and simultaneously avoid overestimating device metrics based solely on theoretical models.

### Response Time

Response time is used to characterize the dynamic ability of a photodetector to respond to variations in incident optical signals. It is commonly defined by the time required for the photocurrent to rise from 10% to 90% of its steady-state value under pulsed illumination (*τ*_rise_) and the time required to decay from 90% to 10% of the steady-state photocurrent (*τ*_fall_). This definition generally assumes that the optical pulse duration is sufficiently long for the device to reach a steady-state response, and that the temporal evolution of the photocurrent is monotonic without pronounced overshoot, oscillation, or hysteresis.

For emerging photodetectors, the response speed is highly sensitive to measurement conditions, including applied bias, load impedance, continuous-wave (CW) incident optical power, and wavelength. Therefore, it should be characterized at multiple representative CW optical power levels within the linear dynamic range, with all experimental parameters explicitly reported. When rectangular optical pulses and 10%–90% rise/fall times are used to evaluate the response speed, it is necessary to verify the monotonicity of the transient photocurrent and confirm that a steady-state plateau is reached after the pulse edges (typically defined as variations of less than 1%), and that this steady-state response is consistent with the CW responsivity. Speed metrics obtained from optical impulse excitation or modulated illumination, such as decay time constants and 3 dB bandwidths, likewise require validation of linear scaling relationships. It should be emphasized that the equivalence among *τ*_rise_, *τ*_fall_, *τ*_*r*,*δ*_, and different definitions of the 3 dB bandwidth holds only when the photocurrent dynamics can be described by a linear first-order model. This assumption is often invalid for emerging photodetectors due to carrier trapping and nonlinear transport effects, and unverified use of such relations may lead to significant errors. Consequently, for general performance benchmarking, small-signal sinusoidally modulated illumination or rectangular pulse measurements are recommended, whereas for application-specific scenarios, the response speed should be characterized under excitation conditions that closely match the actual operating environment.

## Band Structural Engineering

The rich library of 2D materials offers an almost unlimited parameter space for band modulation [115, 116]. Different classes of 2D materials ranging from zero-bandgap semimetals such as graphene to semiconductors like transition metal dichalcogenides possess intrinsic electronic structures (e.g., bandgap type, band-edge positions, carrier effective masses) that dictate the band alignment and interfacial physics when integrated with 3D materials such as Si, Ge, or GaN [55, 117]. Consequently, optimization strategies for band engineering are highly dependent on the selected 2D material system. The semimetallic properties can be exploited to construct efficient Schottky junctions, enabling ultrafast carrier extraction [118–122]. In addition to this, mechanisms such as quantum tunneling and hot-electron effects open opportunities for photodetection beyond the traditional semiconductor bandgap limit [123, 124]. Meanwhile, 2D semiconductors offer a broader range of material options and diverse device architectures for photodetector design [108, 125]. Tailoring band alignment in accordance with the intrinsic properties of different 2D materials thus represents a fundamental pathway toward realizing high-performance and multifunctional photodetectors. This section begins from the materials perspective (2D semimetal and semiconductor) and summarizes the strategies of band modulation in 2D/3D heterojunctions.

### Heterojunctions Based on 2D Semimetals

2D semimetallic materials are widely used in broadband photodetection due to their gapless band structure, high mobility, and inherent topological protections [126–128]. However, 2D semimetal-based photoconductive devices have large noise currents and moderate responsivity due to their gapless properties and weak-light absorption [129, 130]. The integration of 2D semimetals with 3D materials provides additional degrees of freedom for device optimization. vdW heterostructures can improve light absorption and enable built-in electric fields at the interface, which are beneficial for photocarrier separation and transport, potentially leading to improved photoresponse characteristics.

At present, large-area fabricable 2D semimetallic materials, including Gr, MoTe_2_, PtSe_2_, and PdSe_2_, have enabled the realization of high-performance room-temperature infrared photodetector arrays. Wan et al. proposed a self-powered Gr/Si photodetector, in which an ultra-shallow junction architecture effectively addresses the issues of shallow penetration depth and severe carrier recombination in silicon in the ultraviolet spectral region [131]. Benefiting from this design, the device achieves a high responsivity of 0.2 A W^−1^, an ultrafast response time of 5 ns, and an internal quantum efficiency exceeding 100% under zero-bias operation. Tian et al. further investigated carrier multiplication mechanisms arising from interface states at the Gr/semiconductor junction and demonstrated a Gr/GaAs photodetector based on an interface-state-induced gain mechanism. This device exhibits an exceptionally high responsivity of up to 1321 A W^−1^ and a photoconductive gain exceeding 250. Distinct from the aforementioned-studies, this work highlights the critical role of interface states in trapping photogenerated holes and regulating Fermi-level alignment, thereby enabling pronounced gain enhancement [132].

**Fig. 4:**
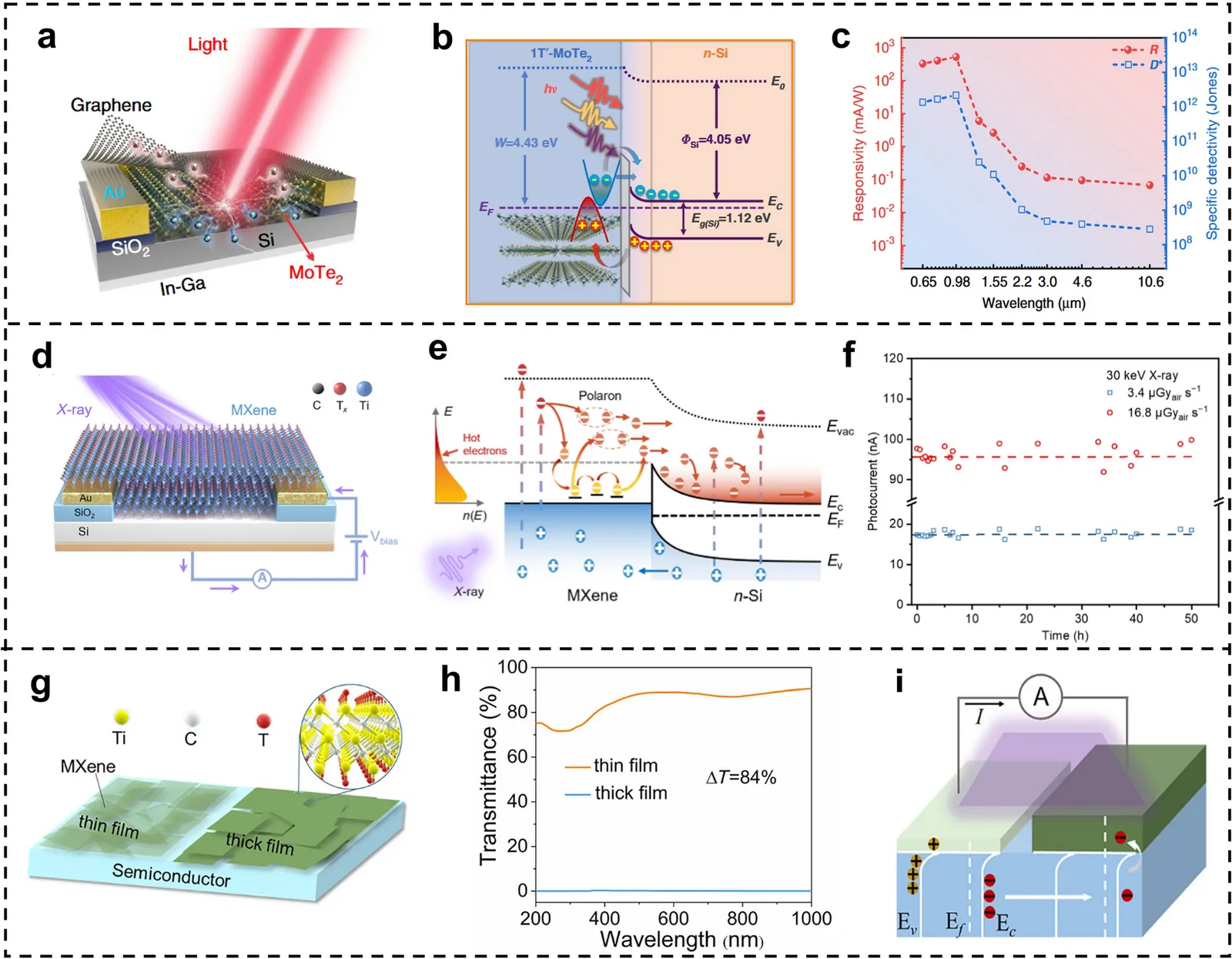
Heterojunctions based on 2D semimetals. **a, b** Schematic illustration and energy band diagram of a graphene/1Tʹ-MoTe_2_/Si Schottky junction device. **c** Responsivity and specific detectivity of the graphene/1Tʹ-MoTe_2_/Si Schottky junction photodetector over a broad spectral range. **a–c** Reprinted with permission from Ref. [62]. Copyright (2023), Springer Nature. **d, e** Structure and simplified band diagram for the operation mechanism of the MXene/Si photodetector. **f** Stability of MXenes/Si detector under X-ray irradiation. **d-f** Reprinted with permission from Ref. [124]. Copyright (2024), John Wiley and Sons. **g** Schematic of the general device structure with MXene electrodes. **h** Transmittance spectra of spin-coated and drop-casted MXene films. **i** Schematic diagram of the band structure and carrier transfer process at the MXene/GaN Schottky junctions under UV illumination. **g–i** Reprinted with permission from Ref. [137]. Copyright (2024), John Wiley and Sons

The 1 T′ and Td phases of MoTe_2_ exhibit metallic or semimetallic characteristics and host exotic physical phenomena such as type-II Weyl semimetal states and superconductivity [131, 132]. Wu et al. achieved thickness-controlled growth of wafer-scale two-dimensional MoTe_2_ via a thermally assisted tellurization process. The resulting Schottky junction devices (Fig. 4a) extend the photoresponse wavelength to 10.6 μm at room temperature. As illustrated in Fig. 4b, electrons can be injected into Si through a thermionic emission mechanism, giving rise to pronounced photoresponses in the near-infrared to mid-infrared spectral range. For infrared photons with energies below the Schottky barrier, photogenerated carriers can directly tunnel through the barrier via an ultrathin insulating layer with a thickness of approximately 4 nm. Consequently, the device exhibits a high specific detectivity over the 3.0–10.6 μm range, reaching values as high as 4.75 ~ 2.8 × 10^8^ Jones (Fig. 4c) [62].

In the infrared spectral range, semimetallic materials can effectively absorb photons through interband transitions across small energy gaps, thereby enabling broadband infrared photodetection. Furthermore, under X-ray irradiation, semimetals can directly generate a large number of nonequilibrium carriers via inner-shell electron excitation and hot-electron effects. Owing to their excellent electrical conductivity and relatively long hot-carrier lifetimes, semimetal/semiconductor Schottky heterojunctions not only enable efficient carrier collection but also effectively suppress dark current, thus achieving a favorable combination of high sensitivity and fast response for X-ray detection. Taking MXene/Si Schottky detectors as a representative example (Fig. 4d), such devices exhibit a high sensitivity of up to 1.2 × 10^7^ μC Gy air^−1^ cm^−2^ and an ultralow detection limit of 2.85 nGy air s^−1^ at room temperature, together with a fast response on the microsecond timescale [124]. The performance enhancement is primarily attributed to hot carriers generated by high-energy radiation crossing the Schottky barrier, as well as the highly efficient carrier transport properties of MXene.

2D semimetals, with their gapless electronic structures, high carrier mobility, and van der Waals interface characteristics, can serve as efficient electrode materials, facilitating reduced contact resistance and suppressed interfacial recombination [49, 62, 133]. MXene, in particularly, combines high electrical conductivity, optical transparency, and solution processability, offering significant advantages for large-area and low-cost device fabrication [134–136]. By tuning the transmittance of MXene layers deposited on semiconductor surfaces, asymmetric photocarrier dynamics across two Schottky junctions can be engineered, thereby enhancing photocurrent responses. As shown in Fig. 4g, Ma et al. deposited MXene films with varying transmittance on semiconductor surfaces. The resulting asymmetry in carrier generation and transport between the two Schottky junctions induces a pronounced enhancement of the photocurrent (Fig. 4h, i). This strategy has been demonstrated to be universal for first- to third-generation semiconductors, including Si and GaAs, and can be realized through an all-solution processing approach, avoiding the need for expensive deposition equipment and harsh vacuum conditions [137].

**Fig. 5:**
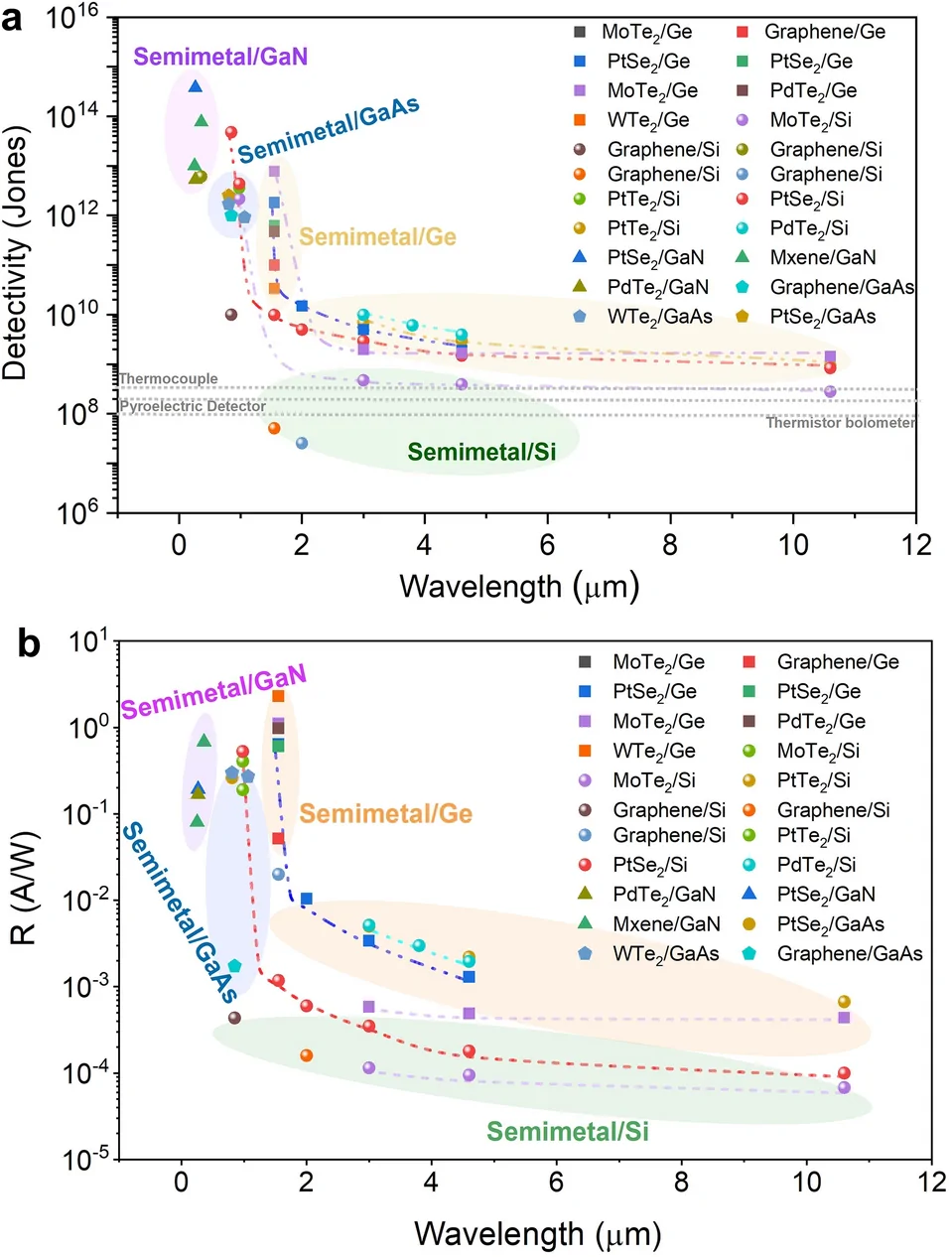
Overview of the performance metrics of 2D semimetal/3D heterostructure photodetectors. **a** Plot of various photodetector technologies against their corresponding detectivity. **b** Response rate comparison of reported 2D/3D photodetectors

To more comprehensively evaluate the performance of these devices and to facilitate direct comparison with reported 2D semimetal/3D heterojunction photodetectors, Fig. 5 summarizes key metrics, including responsivity (Fig. 5a) and detectivity (Fig. 5b), over a broad spectral range. These results not only underscore the critical role of material combinations in determining device performance but also provide valuable guidance for future device optimization and spectral [75, 129, 130, 138–147].

### Heterojunctions Based on 2D Semiconductors

**Fig. 6:**
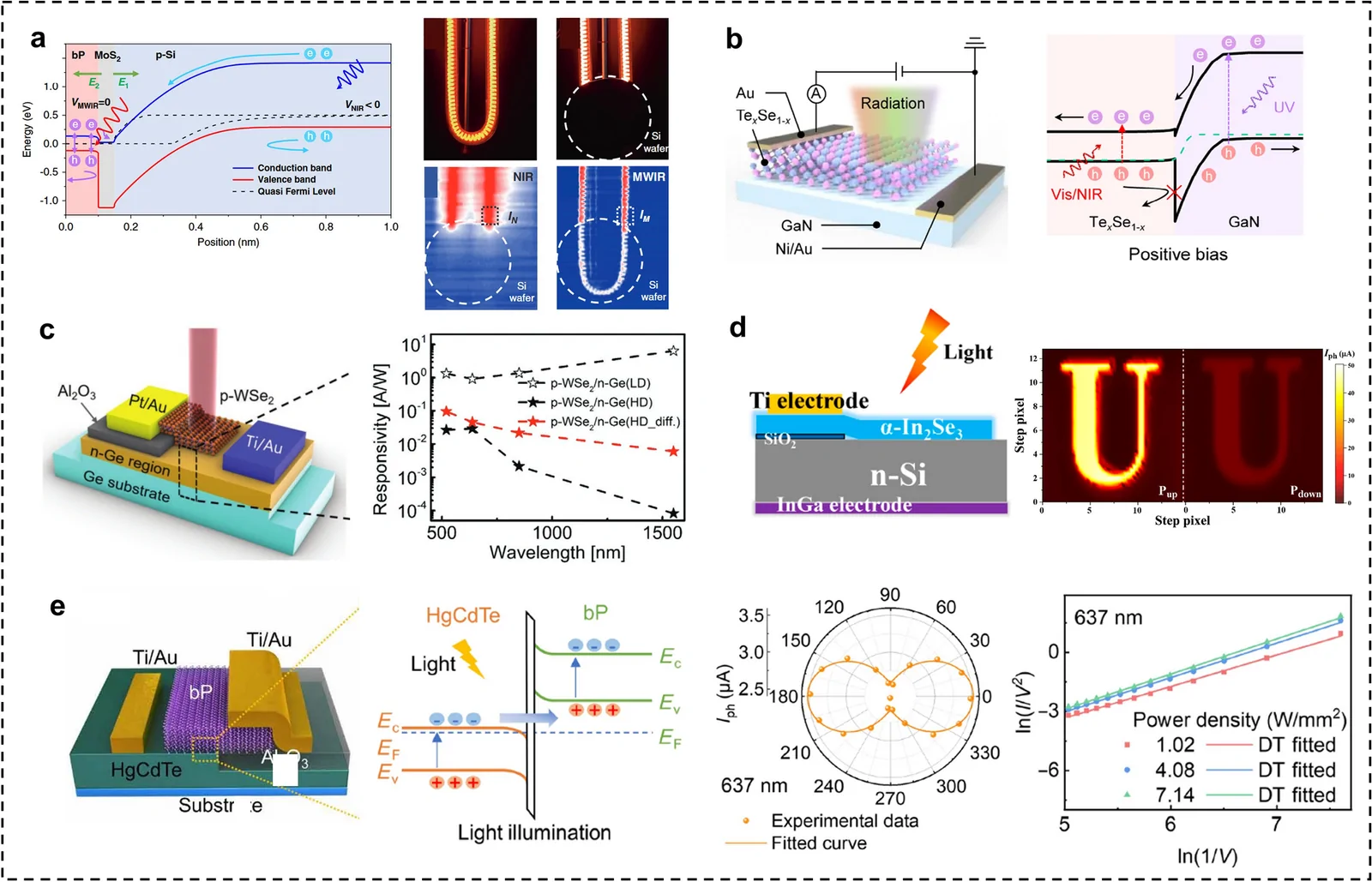
Heterojunctions based on 2D semiconductors.** a** Simulated energy band diagram and electric-field distribution of BP/MoS_2_/Si vdW two-color infrared detector (left). Temporal-spatial coexisting two-color IR imaging (right). Reprinted with permission from Ref. [66]. Copyright (2022), Springer Nature. **b** Structure and energy band diagrams for the device under positive bias. Reprinted with permission from Ref. [37]. Copyright (2025), John Wiley and Sons. **c** Schematic illustration of the WSe_2_/Ge heterostructure photodiode and responsivity over a broad spectral range. Reprinted with permission from Ref. [79]. Copyright (2021), John Wiley and Sons. **d** Device structure for the device. Reprinted with permission from Ref. [151]. Copyright (2023), American Chemical Society. **e** Schematic and energy band diagrams of the HgCdTe/bP heterojunction. The right panel displays the DT fitting curve and polarization-dependent photocurrent under 637 nm laser excitation. Reprinted with permission from Ref. [152]. Copyright (2022), The American Association for the Advancement of Science

The type of band alignment plays a key role in determining carrier separation efficiency and photodetection performance. In type-I (straddling-gap) heterojunctions, the conduction band minimum and valence band maximum of one material are both located within the bandgap of the other material [148]. This leads to the accumulation of photogenerated electrons and holes in the same material, limiting carrier separation efficiency and constraining overall optoelectronic performance [81, 149]. Nevertheless, such structures may be suitable for wavelength-selective detection that requires high internal quantum efficiency [65]. Wu et al. integrated bP and MoS₂ with Si to construct a back-to-back diode, where the built-in electric field enables temporal and spatial separation of near-infrared and mid-wavelength infrared signals. The resulting two-color photodetector achieves a specific detectivity of 6.4 × 10^9^ Jones at 3.5 μm and exhibits low crosstalk (~ 0.05%) for multispectral infrared imaging (Fig. 6a) [66]. Considering the limitations of multi-junction structures on material thickness, doping, and band alignment design, Liu et al. proposed a bias-tunable multispectral photodetector based on a GaN/Te_x_Se_1−x_ heterojunction with a unidirectional barrier (Fig. 6b). This design suppresses dark current (down to ~ 10^–12^ A), controls carrier transport pathways, and enables a spectral response ranging from single-band UV to broadband under different bias conditions [37].

In contrast, type-II (staggered-gap) heterojunctions enable spatial separation of electrons and holes through staggered band alignment, which effectively facilitates carrier extraction and suppresses recombination [150]. As a result, type-II heterojunctions typically exhibit high responsivity, high-speed, and broadband spectral response. Lee et al. reported a high-speed broadband photodiode based on a WSe_2_/Ge heterojunction, in which the heterostructure exhibits a type-II staggered band alignment (Fig. 6c). They further demonstrated that rationally engineering the doping concentration of the Ge layer to optimize the depletion width can maximize the separation and collection of photogenerated carriers, thereby enhancing optical response, light absorption, and photocurrent generation [79]. Jia et al. proposed a self-powered photodetector constructed by integrating a 2D ferroelectric material, α-In_2_Se_3_, with a Si substrate (Fig. 6d). The cooperative effect between the ferroelectric polarization field and the built-in electric field of the heterojunction enhances the photoresponse, while enabling dynamically tunable responsivity with a modulation ratio of up to 10^5^. The device exhibits high responsivity, high specific detectivity (1.6 × 10^13^ Jones), and fast response speed (12 μs) over a broadband spectral range from 405 to 980 nm [151].

Type-III (broken-gap) heterojunctions feature a discontinuous band alignment, in which the conduction band and valence band do not overlap, resulting in a relatively high interfacial barrier. Although such a configuration is unfavorable for conventional carrier transport, ultrafast charge transfer can be achieved via tunneling processes, making type-III heterojunctions attractive for high-speed photodetection and negative differential resistance devices. Jiao et al. constructed a HgCdTe/bP vdW heterojunction with type-III band alignment (Fig. 6e) [152]. The anisotropic layered crystal structure of black phosphorus endows the device with polarization-sensitive photodetection over a broad spectral range from the visible to the mid-wave infrared. Moreover, the linear relationships with positive slopes observed in the ln(1/*V*)-ln(*I*/*V*^2^) fitting curves under different illumination powers indicate the presence of direct tunneling in the heterojunction under light excitation. Through this direct tunneling process, carriers can be rapidly transported across the interface, yielding a high photocurrent and low dark current. As a result, the detector maintains a high detectivity even at room temperature and under zero external bias, with a peak blackbody detectivity of up to 7.93 × 10^10^ Jones.

The advantage of 2D/3D vdWs heterojunctions lies in their ability to achieve controllable band alignment at atomically flat interfaces through delicate band engineering, thereby enabling efficient regulation of photogenerated carrier generation, separation, transport, and recombination processes. However, device performance is also highly dependent on material quality and interface optimization. Han et al. demonstrated that controlling the thickness of 2D films is critical for managing surface and bulk defects, which has a pronounced impact on carrier dynamics and device performance [132]. When the film is too thin, excessive surface defects tend to trap photogenerated carriers, leading to performance degradation. In contrast, overly thick films may introduce a large number of bulk defects, which hindering carrier transport and weakening photovoltaic performance.

## Interface Engineering

Although band structure design serves as a fundamental means of modulating device performance, in practical devices, interface issues cannot be ignored. In conventional semiconductor junctions, interface defects and impurities introduce localized charges that act as Coulomb scattering centers. These charges not only lead to Fermi-level pinning but also enhance carrier scattering and recombination, thereby increasing the dark current [66]. In 2D/3D heterojunctions, the interfacial barrier height, defect-state density, and local field distribution dictate the efficiency of photoelectric conversion. These factors directly influence dark current, carrier recombination, and injection/transport efficiency [151, 152]. Therefore, interface engineering including interlayer insertion, passivation and electrostatic modulation represents a pivotal strategy for performance enhancement.

**Fig. 7:**
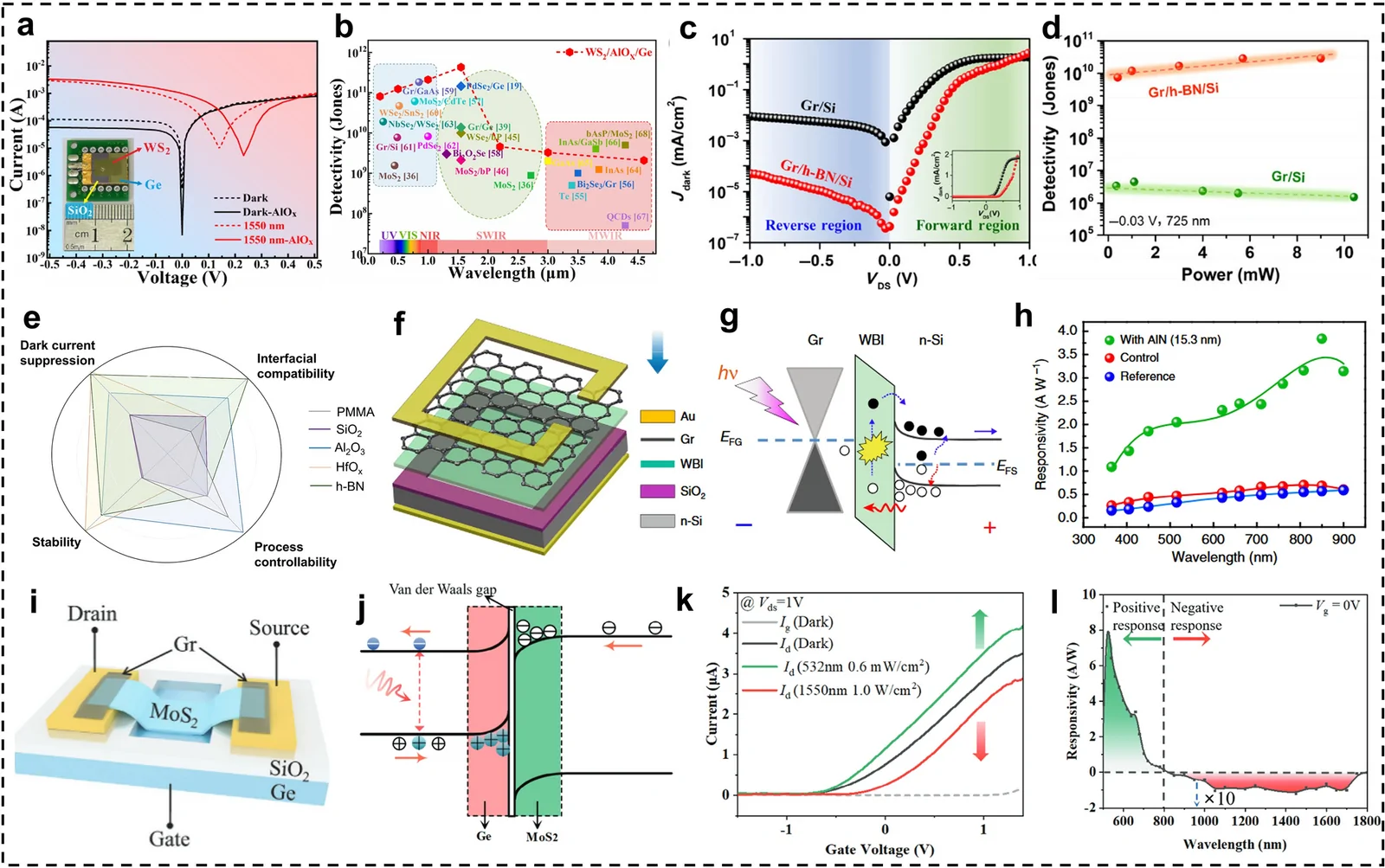
Impact of interface engineering on device performance. **a** I-V curves of the WS_2_/Ge heterojunction device with and without an AlO_x_ passivation layer in the dark and under light illumination (1550 nm). **b** Comparison of D* of the WS_2_/AlO_x_/Ge device with previously reported IR photodetectors. **a, b** Reprinted with permission from Ref. [108]. Copyright (2021), American Chemical Society. **c** Comparison of J_dark−_V_DS_ characteristics of Gr/Si and Gr/h-BN/Si in dark illumination condition. **d** Comparison of detectivity between Gr/h-BN/Si and Gr/Si photodetector at various laser power at 725 nm. **c, d** Reprinted with permission from Ref. [154]. Copyright (2020), Springer Nature. **e** Comparison of interfacial passivation performance among different materials. **f, g** Schematic and corresponding energy band diagram of the fabricated wafer-scale graphene/insulator/Si heterojunction photodetectors. **h** Spectra-dependent photocurrent responsivity of the graphene/insulator/Si photodetector at a bias of − 10 V, comparing with the control and reference devices. **f–h** Reprinted with permission from Ref. [77]. Copyright (2021), Springer Nature. **i, j** Schematic diagram and energy band diagram of the MoS_2_/Ge JFET structure. **k, l** Transfer curves and bipolar photoresponse spectra of the MoS_2_/Ge JFET. Reprinted with permission from Ref. [71]. Copyright (2024), John Wiley and Sons

Sinha et al. reduced the interface defect-state density by cleaning the Si surface and exploiting the vdW contact characteristics of graphene, thereby suppressing Coulomb scattering and Fermi-level pinning [153]. As a result, the interfacial recombination current was reduced, the ideality factor approached unity (*η *≈ 1), non-ideal recombination/scattering mechanisms were minimized, and nearly ideal Schottky rectification characteristics were achieved. Wu et al. introduced a ~ 3 nm AlO_x_ native oxide layer at the WS_2_/Ge interface as a passivation medium [108]. Under dark conditions, the WS_2_/AlO_x_/Ge heterojunction exhibited pronounced rectifying behavior. In contrast, devices without the AlO_x_ interfacial layer showed inferior diode characteristics, with higher reverse current and lower forward current (Fig. 7a). The AlO_x_ layer reduced the interfacial trap-state density and enhanced the built-in electric field, thereby enabling stable self-powered detection at zero bias, highlighting the critical role of interface engineering in achieving low-power photodetection. As shown in Fig. 7b, the device possesses a high specific detectivity in the detection range of 200 nm to 4.6 μm, which is higher than that of other 2D material-based IR photodetectors. Apart from AlO_x_ [139–141] or h-BN, SiO_2_ [86, 142], PMMA [143], and HfO_x_ [144] are also commonly employed as interfacial passivation materials.

Moreover, h-BN with atomically flat surface and superior passivation properties, is particularly suitable for 2D/3D vdW interfaces. Won et al. introduced an ~ 5 nm h-BN tunneling layer at the Gr/Si Schottky interface to achieve selective regulation of carrier injection [154]. Owing to the negative electron affinity of h-BN, it forms a high barrier for electrons while providing a low-barrier pathway for holes, thereby effectively suppressing the dark current and enhancing the injection efficiency of photogenerated holes (Fig. 7c). Furthermore, the interface passivation effect of h-BN effectively mitigates interfacial coupling and reduces defect-assisted recombination. Compared with devices lacking an interfacial passivation layer, the photocurrent-to-dark-current ratio (NPDR) and detectivity are significantly enhanced (Fig. 7d). However, the response speed was limited to ~ 0.9/1.1 ms due to the carrier trapping lifetime within the tunneling layer. Figure 7e compares the interfacial passivation performance of several materials. The interfacial passivation layer can not only serve as a passivation medium to suppress static charge transfer but also construct a tunneling structure that potentially enables impact ionization, leading to photocurrent multiplication. In tunneling heterostructures, the dominant ultrafast quantum tunneling process, rather than carrier drift in the depletion region, is expected to further enhance the response speed [155–160]. As depicted in Fig. 7f, g, Yin et al. proposed to introduce an AlN wide-band insulating layer at the graphene/Si interface [77]. The introduced insulating layer effectively suppresses the dark current of the device. Meanwhile the impact ionization of hot carriers from graphene and minority carriers from Si in the tunneling layer leads to significant photocurrent multiplication. The device exhibits a markedly enhanced responsivity across a broad spectral range compared with the conventional structure and the control device, achieving a responsivity of 3.96 A W^−1^ and a D* of 1.13 × 10^8^ Jones at 850 nm (Fig. 7h).

The atomically flat and weakly coupled interfaces in vdW heterojunctions provide a unique platform for the fine regulation of interlayer charge interactions. In particularly, under specific band alignment conditions, the spatial separation of interfacial charges does not necessarily weaken their interaction. Instead, coulomb coupling can still play a crucial role in governing carrier dynamics and modulating the polarity of the photoresponse [161, 162]. As shown in Fig. 7i, j, You et al. investigated the formation of interlayer Coulomb interactions at the vdW interface by utilizing the vdW contact between MoS₂ and Ge and its energy band alignment [71]. Specifically, due to the Fermi energy-level alignment, electrons tend to accumulate on the MoS_2_ side and holes tend to accumulate on the Ge side. This energy band alignment ensures that photogenerated carriers have preferred paths at the interface when photons of different wavelengths are absorbed. Visible light is mainly absorbed by MoS_2_, which allows the photogenerated electrons to be injected into the channel smoothly, resulting in a positive photoresponse, near-infrared light is mainly absorbed by Ge, which leads to a large number of hole accumulations at the interface. The accumulated holes at the interface reduce the electron density in the MoS_2_ channel through coulombic attraction, thus inhibiting the channel electron transport and resulting in a negative optical response (Fig. 7k, l). The photoluminescence spectrum measured at 77 K under 532 nm excitation further confirms the Coulomb interaction mechanism.

## Electrical Coupling

**Fig. 8:**
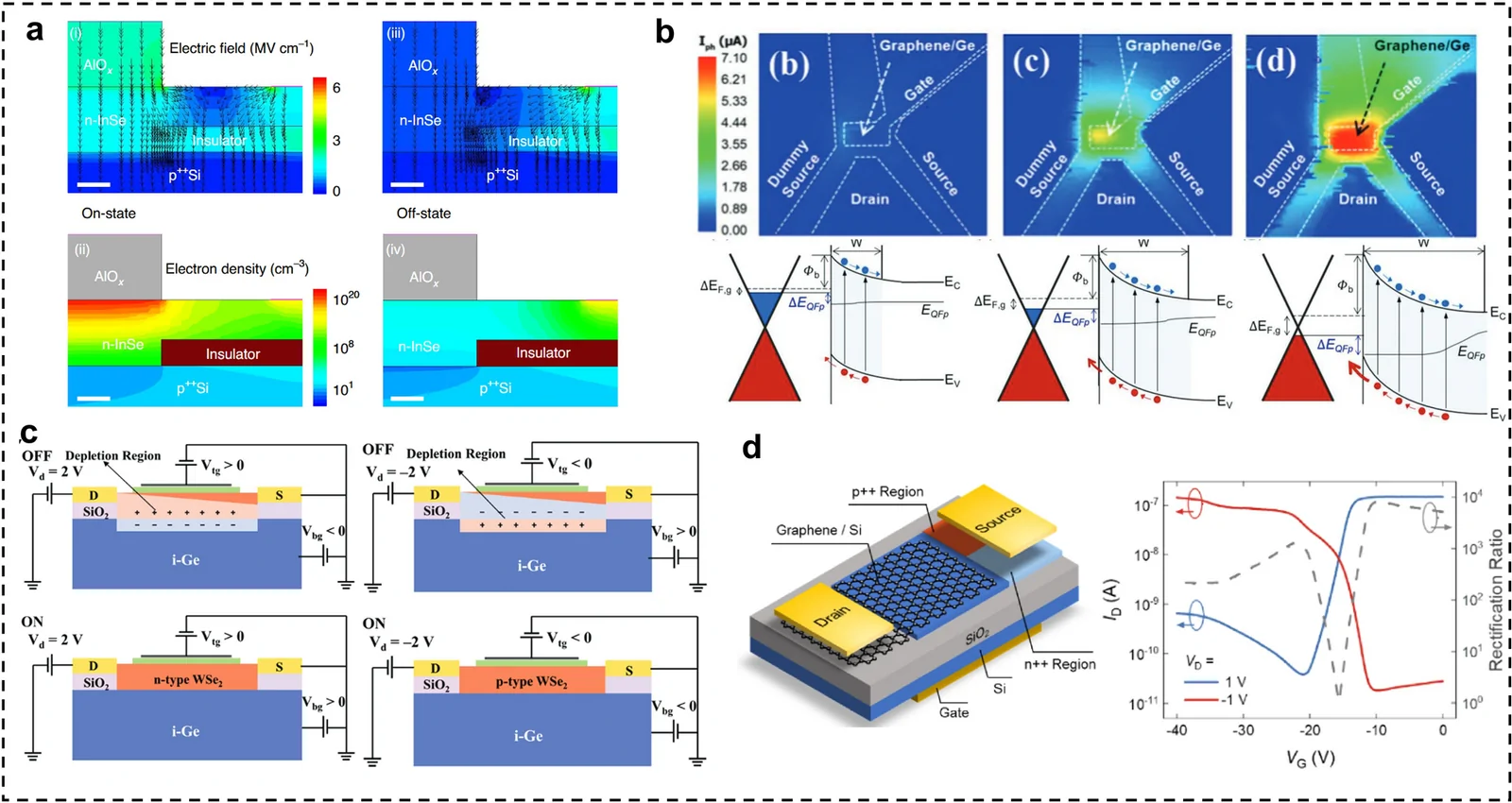
Modulation of device performance by electric fields. **a** TCAD-simulated electric-field contour across the InSe/Si HJ-TTs in the on (i) and off (iii) state. The electron-density profile for the simulated InSe/Si HJTTs in the on (ii) and off (iv) state. Reprinted with permission from Ref. [165]. Copyright (2022), Springer Nature. **b** Scanning photocurrent mapping and energy band diagrams of the graphene/Ge photodetector taken at V_G_ = 10, 0, and − 10 V with an illumination of 1550 nm, 12.9 μW, respectively. Reprinted with permission from Ref. [168]. Copyright (2019), John Wiley and Sons. **c** Schematic illustration of the working mechanism of the nJFET and pJFET. Reprinted with permission from Ref. [105]. Copyright (2022), John Wiley and Sons. **d** Schematic and transfer characteristic curves of the graphene/Si Schottky diode. Reprinted with permission from Ref. [73]. Copyright (2022), American Chemical Society

In addition to modulating carrier transport and photodetection efficiency through energy-level alignment and defect/passivation control, electrostatic modulation via electric-field coupling offers a complementary and highly versatile strategy. By applying gate or local electric fields, the carrier density, injection, and transport in the heterojunction can be dynamically tuned, representing an effective approach for the development of multifunctional photonic systems. The atomically thin nature of 2D materials enables strong electrostatic control, facilitating smaller subthreshold swings (SS) and higher on-state current densities, thereby enhancing the on/off ratio. In addition, the intrinsic self-passivating property of 2D vdW materials allows easy integration with metal gates and thin dielectric layers, further strengthening electrostatic modulation [163, 164]. In principle, the atomically sharp interfaces of vdW heterostructures are particularly advantageous for tunneling devices, which are highly sensitive to impurities and interface defects [90]. For example, Miao et al. reported a 2D InSe/3D Si heterojunction tunneling transistor, where the ultrathin nature of the 2D material combined with the high doping concentration of the 3D material enabled precise band alignment tuning via gate voltage, achieving a favorable balance between ultralow-power consumption (SS as low as 6.4 mV dec^−1^) and high drive current (on/off ratio ~ 10^6^), as shown in Fig. 8a [165]. In the field of photodetection, electric-field coupling can optimize the separation and transport efficiency of photogenerated carriers by modulating the Schottky barrier and depletion region, with the key being the balance between photocurrent gain and dark current suppression [111, 165–167]. Chang et al. employed a transparent ZnO top gate to modulate the barrier height of a Gr/Ge Schottky junction, achieving dark currents on the order of μA cm^−2^ while reaching a high responsivity of 70 A W^−1^, approximately five times higher than comparable commercial devices. This configuration also extends the spectral response into the near-infrared range, with a fabrication process far simpler than that of conventional Ge photodetectors [166]. Furthermore, by strategically modulating the Schottky barrier height at the Gr/Ge junction using a transparent ZnO top-gate electrode, the photoresponse was extended into the near-infrared region, while the fabrication process remained much simpler than that of commercial Ge photodetectors (Fig. 8b) [168].

The modulation of optoelectronic performance via electric-field coupling highlights its great potential in multifunctional photonic systems. Wang et al. realized a MoS_2_/Ge JFET in which the response under 532 nm illumination is dominated by MoS_2_ absorption, achieving a responsivity of approximately 66 A W^−1^. Under 1550 nm illumination, however, the photothermal effect in MoS_2_ competes with the photogenerated carriers in Ge, resulting in a non-monotonic dependence of the photoresponse on gate voltage and light intensity [55]. Yang et al. leveraged the ambipolar nature of WSe_2_ in a WSe_2_/Ge JFET, using the top gate to switch between n-type and p-type operation, achieving near-ideal switching characteristics (SS ≈ 60 mV dec^−1^, on/off ratio ≈ 10^5^), as shown in Fig. 8c. Building on electric-field coupling for performance modulation, Chen et al. precisely controlled the carrier type and local built-in field in Si via a back gate, enabling in-sensor array-based image convolution and pseudo-binary multiply-accumulate (MAC, a basic arithmetic operation that accumulates the result of a multiplication)) operations, which significantly improved BNN inference accuracy from 77.04% to 98.35%. (Fig. 8d) [73].

**Fig. 9:**
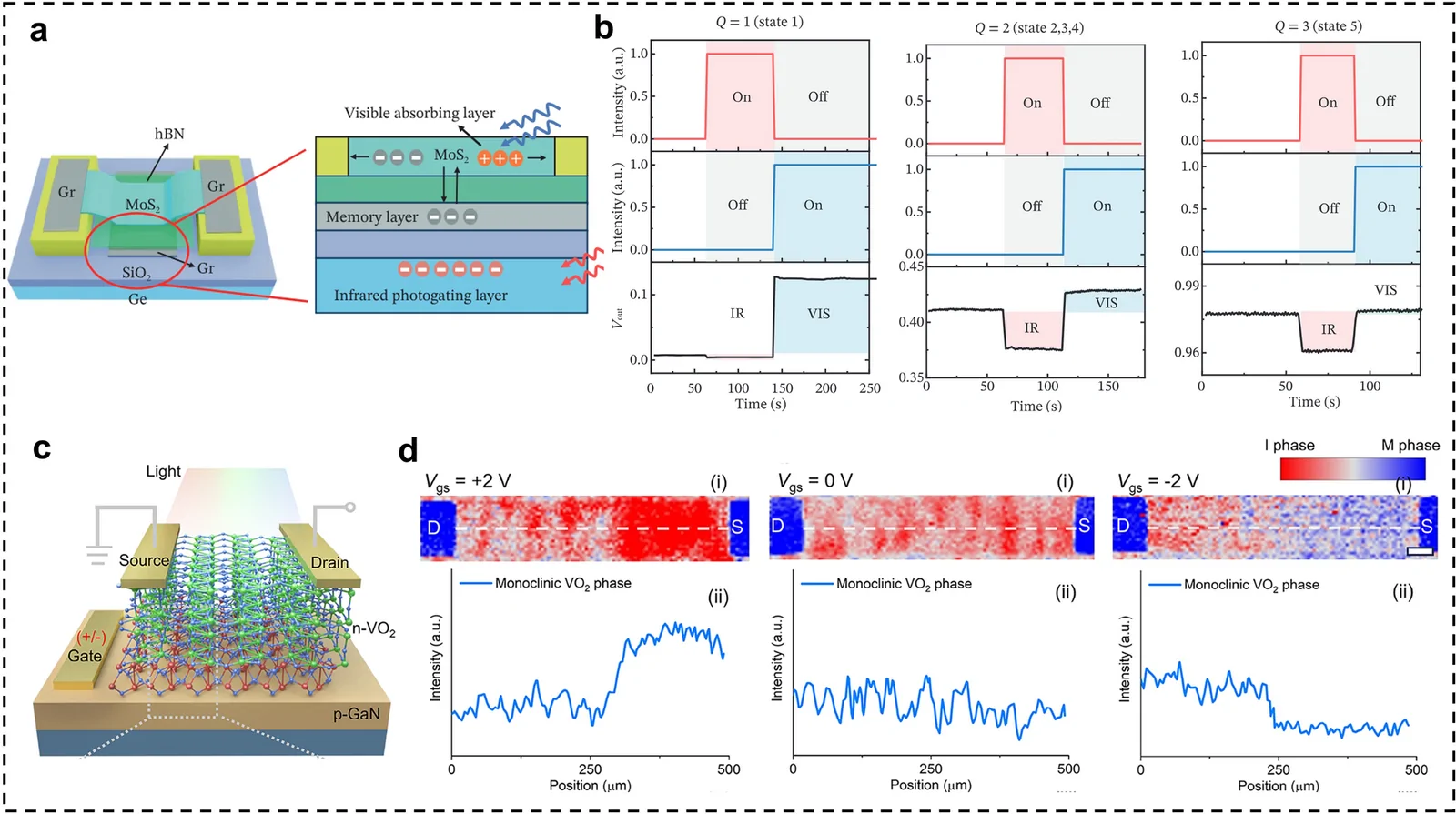
Dynamic gate voltage modulation of carrier distribution and photodetection states. **a** Schematic of the FG-PT with an extra Ge gate (left) and schematic diagram of device working mechanism (right). **b** Output voltage and visible–infrared light response under different programming states: Q = 1 corresponds to the programmed state, Q = 2 corresponds to the intermediate state, and Q = 3 corresponds to the erased. Reprinted with permission from Ref. [70]. Copyright (2025), John Wiley and Sons. **c** Schematic of the PTE device with n-VO_2_ film epitaxial growth on the p-GaN layer to form a p–n junction. **d** Raman mapping images (ⅰ) for phase distribution along the drain and source regions, corresponding mapping section profile (ⅱ), and schematic diagram (ⅲ) at different states when the gate voltage is + 2 V (left), 0 V (center) and − 2 V (right), respectively. Reprinted with permission from Ref. [169]. Copyright (2025), John Wiley and Sons

The gate in 2D/3D devices can act not only as a conventional FET control terminal but also as a nonvolatile memory element or phase-change switch, enabling programmability and in-sensor computing. Wang et al. demonstrated a MoS_2_/Gr/Ge floating-gate phototransistor (FG-PT) for wavelength-selective imaging from visible to near-infrared. The graphene floating gate stores charges to modulate the MoS_2_ channel, switching among visible, dual visible-infrared, and infrared imaging modes, while the 3D Ge substrate provides near-infrared absorption and grating-induced current modulation, enabling broadband imaging. (Fig. 9a, b) [70]. Li et al. used a VO_2_/GaN three-terminal photothermoelectric (PTE) detector, where gate voltage induces a local VO_2_ insulator–metal phase transition, enabling bipolar PTE response. This approach allows hardware-level convolution emulation and image preprocessing, with high-quality GaN interfaces ensuring epitaxial VO_2_ uniformity, promising for large-scale integration and edge computing. (Fig. 9c, d) [169].

## Geometrical/Structural Engineering

**Fig. 10:**
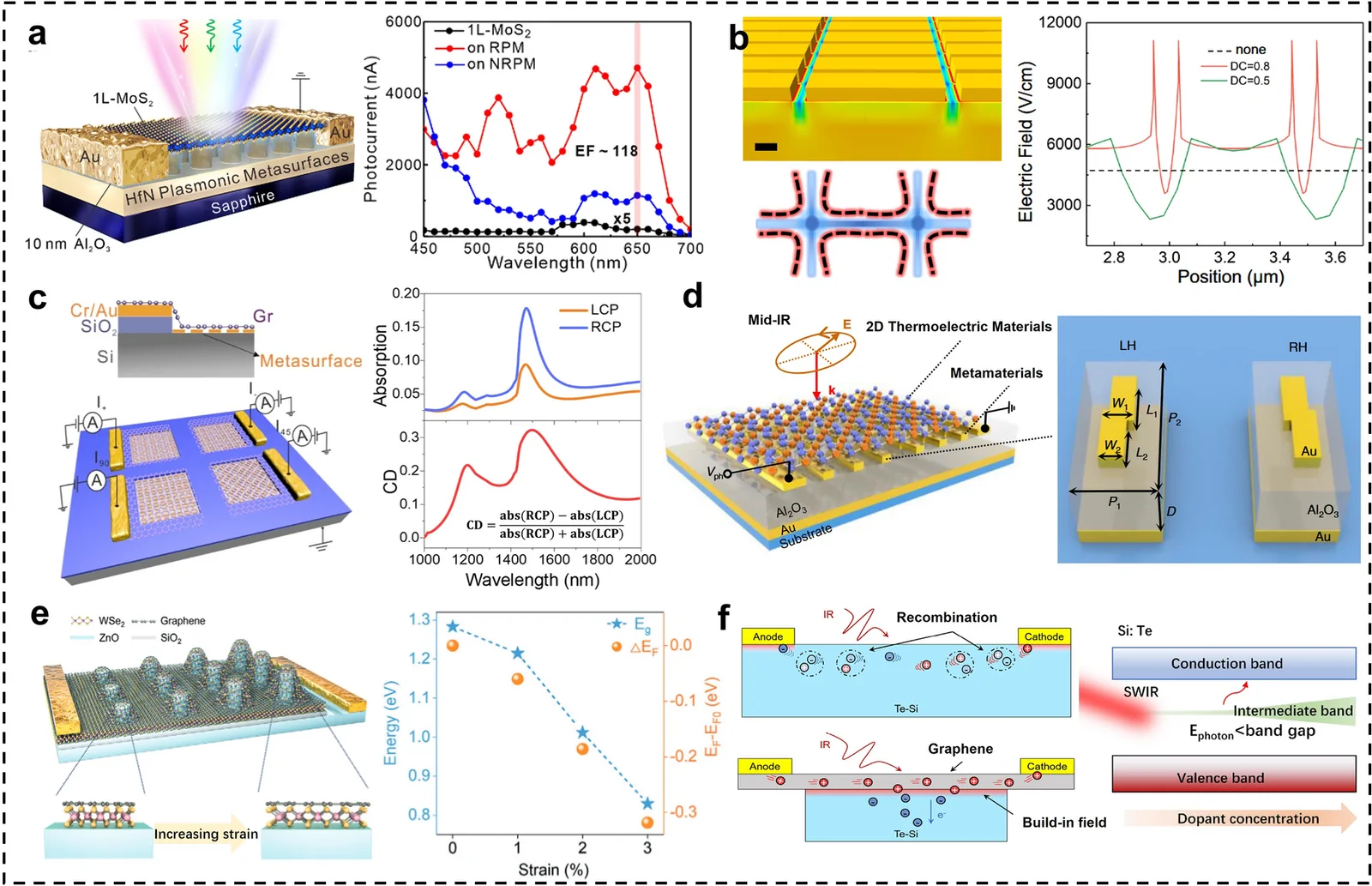
The impact of topological structural design on device performance. **a** Schematic and measured spectral photocurrent of plasmonic PDs. Reprinted with permission from Ref. [174]. Copyright (2022), American Chemical Society**. b** Simulated potential distribution of the slit structure in cross-sectional and longitudinal sections. Reprinted with permission from Ref. [88]. Copyright (2024), Springer Nature. **c** Schematic diagram and calculated optical absorption spectra of the proposed plasmonic polarimeter. Reprinted with permission from Ref. [68]. Copyright (2020), American Chemical Society. **d** Device architecture design for resonance thermoelectric photoresponse. Reprinted with permission from Ref. [48]. Copyright (2024), American Chemical Society. **e** Schematic diagram of the heterostructure with gradient strain modulation. Reprinted with permission from Ref. [110]. Copyright (2024), Springer Nature. **f** Schematic diagram and working mechanism of the graphene/Te-Si 2D/3Dphotodetectors. Reprinted with permission from Ref. [182]. Copyright (2024), John Wiley and Sons

In addition to controlling carrier transport, injection, and photoresponse at the atomic or interfacial scale, the geometric and structural characteristics of 3D materials provide additional degrees of freedom for modulation. By leveraging parameters such as dimensionality, shape, and periodicity, light-matter interactions, carrier dynamics, and device functionalities can be precisely engineered, enabling performance enhancement of 2D/3D heterojunction devices that goes beyond interface optimization alone [170–172]. This paradigm holds promise for overcoming the limitations of conventional devices in optical enhancement, electrical modulation, and functional expansion, thereby driving the development of high-performance, miniaturized, and multifunctional optoelectronic devices. Yang et al. employed inverted conical nanopores to form a graded refractive index structure, which reduced light reflection (< 10%) and enhanced light absorption (> 90%) [173]. Moreover, the conformal contact between graphene and silicon nanopores shortened the carrier transport path and reduced recombination losses. Syong et al. transferred scalable monolayer MoS_2_ onto plasmonic nanostructures to maximize the active area of MoS_2_ photodetectors. Owing to the strong light-matter interaction between monolayer MoS_2_ and HfN resonance plasmonic metasurfaces, a pronounced PL enhancement was observed within the resonance plasmonic metasurfaces (Fig. 10a) [174]. In addition to enhancing absorption, the versatile structural designs of three-dimensional materials can flexibly enable electric-field modulation, dynamically tune the response wavelength, and overcome the fixed bandwidth limitations of conventional devices. In order to excite the wide-spectrum detection capability of graphene while ensuring high responsivity, Jiang et al. introduced 2D dielectric slits induced potential wells into a single-layer graphene photodetector [88]. Simulations of the potential distribution indicate that the surface potential of the all-silicon dielectric remains constant, whereas the periodic slit structure can lead to the generation of valley potential. As the gap spacing decreases, the coupling of edge electric fields becomes stronger, inducing a larger localized electric field and a deeper valley potential, as shown in Fig. 10b. The periodic surface potential wells on the graphene surface, and subsequently generate strong trapping force on photogenerated carriers in graphene spatially (laterally and vertically) and inhibit their recombination, which realizes high responsivity (0.2 ~ 38 A W^−1^) at room temperature. By adjusting the slit orientation, anisotropic structures can be introduced to achieve linearly polarized selective detection.

As a type of artificial micro-nanostructured material, 3D material-based metasurfaces can achieve flexible polarization responses by locally manipulating light. The integration of metasurfaces with 2D materials opens new avenues for developing compact, high-performance polarization detectors in the infrared region [175]. Wei et al. proposed a metasurface-mediated bulk photovoltaic-like (BPVE-like) effect, in which a non-centrosymmetric T-shaped metallic nanoantenna array generates directional photocurrents in graphene, enabling polarization-angle detection without calibration using a single device [176]. Furthermore, Li et al. developed a four-pixel monolithically integrated polarization detector that combines chiral metasurfaces with Gr/Si Schottky junctions. By mathematically reconstructing the four photocurrent signals, full-Stokes detection was realized at the 1550 nm near-infrared wavelength (Fig. 10c**)** [68]. These studies demonstrate that integrating plasmonic metamaterials with polarization-selective field enhancement into semiconductors can produce polarization-sensitive photocurrents. However, most detectors rely on photoconductive or photovoltaic effects, which require matching the resonance wavelength of the plasmonic metamaterials with the semiconductor bandgap [177]. Dai et al. exploited the photothermoelectric effect in 2D thermoelectric materials, allowing device responses to be independent of the active semiconductor’s bandgap, and enabling straightforward operation across visible, near-infrared, and terahertz wavelength ranges (Fig. 10d**)** [48]. Moreover, by adjusting the proportion of left- and right-handed metasurface distributions, polarity reversal can be achieved in linear and circular polarization detection. Dai et al. further proposed a dynamically reconfigurable polarization detector, in which gate voltage modulates the photothermoelectric response of two orthogonal polarization-sensitive regions, enabling switching between different operational modes via electrical control to meet diverse detection requirements [178].

The geometric design of 3D semiconductors not only actively modulates optical responses but also introduces geometric gradients in 2D materials, thereby inducing strain modulation in the 2D layer. Strain modulation enhances the driving force for carrier separation by tuning the band alignment of heterojunctions in 2D optoelectronics, enabling phenomena such as exciton funnels, site-controlled single-photon emission, and the regulation of interlayer exciton behavior [172, 179, 180]. Zeng et al. introduced graded biaxial tensile strain (1.3%–4.0%) into WSe_2_ using ZnO nanorod arrays of varying heights [110]. As the strain in 2D WSe_2_ increased, the photocurrent in the vdWs heterojunction region rose significantly, with the EQE improving from 11.4% to 35.3%. This enhancement was attributed to strain-induced modulation of the WSe_2_ electronic structure, as density functional theory calculations revealed a linear bandgap reduction with strain, leading to stronger indirect bandgap emission (Fig. 10e). In addition to versatile structural designs, the mature doping techniques of 3D semiconductors offer significant advantages for 2D/3D heterojunctions. Precise control over the doping concentration and type in 3D semiconductors enables flexible band engineering, thereby optimizing the built-in electric field and enhancing carrier separation and transport efficiency. For example, heavily doped silicon obtained through ion implantation exhibits advantages such as broadband response, strong infrared absorption, and excellent compatibility with CMOS processes in photodetectors [181]. As shown in Fig. 10f, Jiang et al. proposed integrating graphene with Te-hyperdoped silicon (Te-Si), achieving ultrahigh gain in the SWIR region [182]. The device exhibits a high responsivity of 100 A W^−1^ at 1.55 μm and an ultralow NEP of 0.71 pW Hz^−1/2^ at room temperature, outperforming previously reported Gr/Si photodetectors.

## Comparison of Various Modulation Mechanisms

To facilitate a clear understanding of the literature on photodetection technologies based on 2D/3D heterostructures, Table 2 summarizes the recent advances in photodetection achieved using different modulation strategies. Band engineering represents a fundamental strategy for enhancing the optoelectronic performance of 2D/3D heterostructures. Its core lies in the rational design of band alignment configurations such as Schottky junctions, Type-I/II/III heterojunctions, and unidirectional barriers to systematically optimize the generation, separation, and transport of photogenerated charge carriers. High photocarrier collection efficiency is achieved through appropriate band bending and the formation of a built-in electric field. By effectively suppressing thermally activated carrier injection, this approach reduces dark current, thereby improving the on/off ratio and specific detectivity. Moreover, different band alignment schemes enable multispectral, broadband, and even tunneling-assisted sub-bandgap photoresponse, providing viable pathways to overcome intrinsic bandgap limitations and extend the detectable spectral range.

**Table 2:** Latest advancements in photodetection technology based on 2D/3D heterostructures

Engineering techniques	Structure	Spectral range (μm)	Band structural	Bias (V)	R (A W^−1^)	D* (Jones)	Response time	Optimal performance	Device Scale (pixel area)	References
Band structural engineering	MoTe_2_/Ge	0.2 to 10.6	Schottky junction	0	0.44 × 10^–3^ (10.6 μm)	1.44 × 10^9^ (10.6 μm)	7.6/13.7 ms (1.55 μm)	Self-powered broadband infrared imaging	8 × 8 array (1 × 1 mm^2^)	[49]
	MXene/GaN	0.36	Schottky junction	0	0.081 (360 nm)	6.35 × 10^12^ (360 nm)	31/29 ms (360 nm)	MXene electrodes and transmittance contrast-induced photocurrent	Single device	[137]
	MXene/Si	X-ray	Schottky junction	− 1	–	–	31/35 μs (X-ray)	High-sensitivity (1.2 10^7^ μC Gyair^−1^ cm ^2^) and radiation-stable	Single-pixel imaging	[124]
	Gr/MoTe_2_/Si	0.26–10.6	Schottky junction	0	0.068 × 10^3^ (10.6 μm)	2.8 × 10^8^ (10.6 μm)	1.9/41.5 μs (980 nm)	Wafer-scale phase-controlled vdW growth of MoTe_2_ and ultrabroadband detection	8 × 8 array room-temperature MIR imaging (200 × 200 μm^2^)	[62]
	Gr /Te-Si	2.7	Schottky junction	1	16.3 (2.7 μm)	5.6 × 10^9^ (1.5 μm)	–	Ultrahigh photogain of 10^9^ at room temperature	Single device	[182]
	Gr/PtTe_2_/Si	1–10.6	Schottky junction	0	0.67 × 10^3^ (10.6 μm)	0.93 × 10^9^ (10.6 μm)	2.4/32 μs (808 nm)	van der Waals epitaxial growth of wafer-scale PtTe_2_ for high-performance MIR photodetection	Single-pixel room-temperature MIR imaging	[75]
	PtSe_2_/GaN	0.265	Schottky junction	0	0.19 (265 nm)	3.8 × 10^14^ (265 nm)	45/102 μs (265 nm)	Self-powered deep ultraviolet photodetection	Single device	[138]
	BPQDs/ PdSe_2_/Si	0.78	Schottky junction	0	0.3 (780 nm)	10^13^ (780 nm)	38/44 μs (780 nm)	Wafer-scale growth of high-quality PdSe_2_ films	4 × 4 array	[183]
	Gr/Si	1.55	Schottky junction	10	0.25 (1.55 μm)	–	–	Resonant cavity enhanced	Single device	[130]
	Gr/PdSe_2_/Ge	0.2 to 3.04	Schottky junction	0	0.69 (980 nm)	1.73 × 10^13^ (980 nm)	6.4/92.5 μs (980 nm)	Polarization-sensitive broadband photodetection	Single device	[54]
	Gr/Ga_2_O_3_	0.254	Schottky junction	20	39.3 (254 nm)	5.92 × 10^13^ (254 nm)	–	Deep UV	Single device	[84]
	Te_x_Se_1-x_/GaN	0.365 to 1	Unidirectional barrier	–	0.25 (365 nm) 0.58 (1060 nm)	5.04 × 10^11^ (365 nm) 2.64 × 10^9^ (1060 nm)	–	Spectral response range modulated by bias polarity and magnitude	Single device with bias-tunable multispectral imaging	[37]
	CdS/Gr/Ge	0.45	Sandwich structures	–	− 3376 to 3584	-	40/40 ms (450 nm)	All-Optical modulation and nearly symmetric PPC/NPC	Single device	[69]
	WSe_2_/SiC	0.2 to 1.1	Type I	–	25.7 (275 nm) 3 (830 nm)	3.85 × 10^12^ (275 nm) ~ 10^11^ (830 nm)	42/42 μs (635 nm)	UV–Vis-IR response	Single-pixel imaging	[184]
	Gr/MoSe_2_/ HgCdTe	2.46 to 4.17	Barrier-Type	− 0.1	–	3.01 × 10^10^ (~ 3.1 μm)	65/60 μs	Effective suppression of dark current at elevated temperatures	Single device	[185]
	Ge/MoS_2_/bP	0.633 1.55 2	p-n-p	Bias-switched	–	–	–	Bias-tunable multiband photodetection (VIS, SWIR and MWIR)	Single-pixel multispectral imaging	[67]
	g-C_3_N_4_/GaN	0.365	Type-II	− 0.4	0.58 (365 nm)	2.06 × 10^14^ (365 nm)	0.35/0.9 s (365 nm)	Electron trapping effects by the S-doped sites for gain	Single device	[186]
	Bi_2_Se_3_/GaN	0.25 to 1.05	Type-II	0	0.07 (350 nm)	1.79 × 10^12^ (350 nm)	72/61 ms	Device performance modulated by the thickness of 2D films	Single-pixel broadband imaging	[147]
	WSe_2_/GaN	0.36 0.72	Type I	Bias-switched	–	–	–	Dual-wavelength photoresponse	Single device	[65]
	α-In_2_Se_3_/Si	0.4 to 0.98	Type-II	0	0.026 (P_down_) 0.5 (P_up_)	6.3 × 10^11^ (P_down_) 1.6 × 10^13^ (P_up_)	12/18 μs (P_down_) 43/310 μs (P_up_)	Photoresponse modulated by intrinsic ferroelectricity in α-In₂Se₃	Single-pixel NIR imaging	[152]
	MoS_2_/GaN	365–900	Type I	− 3	28.18 (365 nm)	9.08 × 10^14^ (365 nm)	98/148 ms	Broad spectral response	Single device	[149]
	bP/MoS_2_/Si	0.7 to 4.5	Back-to-back	0	–	6.4 × 10^9^ (3.5 μm)	–	NIR/MWIR two-color infrared imaging	Single-pixel temporal-spatial coexisting two-color imaging	[66]
	WSe_2_/Ge	0.52 1.55	Type I	− 1.5	1.3 (520 nm) 6.4 (1550 nm)	–	3/3 μs (520 nm) 30/5 μs (1550 nm)	Thickness- and doping-concentration modulation of n-Ge	Single device	[79]
	PtSe_2_/GaAs	0.25 to 1.2	Type I	0	0.262 (808 nm)	2.52 × 10^12^ (808 nm)	5.5/6.5 μs (808 nm)	Deep UV to NIR	Single device	[81]
	bP/HgCdTe	1 to 4.5	Type Ⅲ	0	0.168 (4.3 μm)	7.93 × 10^10^ (4.3 μm)	150/110 μs (637 nm)	Polarization-sensitive MIR detection	Single device	[151]
	SnS_2_/SiC	0.325	Type-II	5	2.42 × 10^4^ (325 nm)	7.3 × 10^13^ (325 nm)	17/17 ms (325 nm)	High-performance DUV-VIS photodetection	Single device	[187]
	SnS_2_/Si	0.56	Type Ⅱ	1	3.04 (560 nm)	2.39 × 10^12^ (560 nm)	32/9.8 μs (560 nm)	Broadband photoresponse	Single device	[188]
	WS_2_/Si	0.4 to 1.1	Type Ⅱ	− 2	1.11 (632 nm)	5 × 10^11^ (632 nm)	-	Broadband photoresponse	Single device	[150]
	GQDs/WSe_2_/Si	0.74	–	− 3	0.7 (740 nm)	0.24 × 10^9^ (740 nm)	0.2 ms (740 nm)	Broadband photoresponse	Single device	[189]
	MoS_2_/SiC	0.365	–	20	5.7 (365 nm)	3.07 × 10^10^ (365 nm)	0.6/0.3 s	UV photodetection	Single device	[190]
Interface engineering	Gr/AlN/Si	0.3 to 0.8	Schottky junction	− 10	3.96 (850 nm)	1.13 × 10^8^ (850 nm)	1.9/1.4 ms (365 nm)	Enhanced photocurrent multiplication and decreased dark	Wafer-scale (8 × 8 mm^2^)	[77]
	WS_2_/AlO_x_/ Ge	2.2 to 4.6	Type-II	1	3.1 × 10^–3^ (4.6 μm)	2.08 × 10^9^ (4.6 μm)	9.8/12.7 μs (1550 nm)	Defect engineering extends the spectral response range and interface passivation enhances the optoelectronic performance	Single device	[108]
	Gr/Al_2_O_3_ /Ge	1.5	Schottky junction	2	1.2 (1550 nm)	1.9 × 10^10^ (1550 nm)	–	Increasing the tunneling distance of low-energy carriers and reducing the Fermi-level pinning effect suppress the dark current	Single device	[191]
	PtSe_2_/Al_2_O_3_/Ge	1.5	Type I	0	4.09 (1550 nm)	1.17 × 10^9^ (1550 nm)	32/18.9 μs (1550 nm)	Broadband photoresponse	Single-pixel NIR imaging	[155]
	PtSe_2_/ SiO_2_/Si	0.37 to 2	Type I	0	8.06 (808 nm) 0.65 × 10^–3^ (1550 nm)	4.78 × 10^13^ (808 nm)	14.1/15 μs (808 nm)	Broadband photoresponse	9 × 9 array room-temperature NIR imaging	[192]
	Gr/GdIG/ Si	0.23 to 0.95	Schottky junction	− 2	–	1.35 × 10^13^ (633 nm)	0.1/0.2 ms	Interlayer increases the barrier height and passivates the contact surface	Single device	[193]
	Gr/hBN/Si	0.725	Schottky junction	− 0.03	–	2.83 × 10^10^ (725 nm)	0.9/1 ms (725 nm)	Efficient photovoltaic effect	Single device	[154]
	Gr/SiO_2_/Si	0.89	Schottky junction	0	0.73 (890 nm)	4.08 × 10^13^ (890 nm)	0.3/0.8 ms (890 nm)	High Detectivity	Single device	[107]
Electrical coupling	Ge/Gr/ hBN/MoS_2_	0.532 1.55	Floating gate	0.05 (V_ds_) ± 10 V (V_g_)	12.7 (532 nm) 0.024 (1550 nm)	3.14 × 10^11^ (532 nm) 4.95 × 10⁸ (1550 nm)	–	The floating-gate memory function enables reconfigurable response	Single-pixel imaging	[70]
	Gr/Si	0.685	Schottky junction	1 (V_ds_)	–	–	–	Gate programming of the photoresponsivity and rectification direction	Single device	[73]
	MoS_2_/Ge	0.532 1.55	–	2 (V_ds_)	Changed	5.3 × 10^9^ (532 nm) 4.43 × 10^6^ (1550 nm)	40/160 µs (532 nm) 40/40 µs (1550 nm)	Light and gate modulated photoresponse	Single device	[55]
	Gr/Si	0.39 to 0.85	Schottky Junction	1 (V_ds_) − 15 V (V_g_)	70 (395 nm)	2 × 10^13^ (530 nm)	–	Top gate modulates the Schottky barrier height, enabling enhanced photoresponse gain and suppressed dark current	Single device	[166]
	Gr/Ge	1.55	Schottky Junction	0.5 (V_ds_) − 10 V (V_g_)	0.75 (1550 nm)	0.43 × 10^9^ (1550 nm)	–	Top gate modulates the Schottky barrier height	Single device	[168]
Geometrical/structural engineering	Gr/Si (2D dielectric slit)	0.2 to 11	Schottky junction	0.1	7.5 (11 μm) 38 (1.55 μm)	1 × 10^9^ (blackbody radiation)	–	High-efficiency photodetectors at NIR to MIR and polarization-sensitive detection	Single device	[88]
	MoS_2_/ Al_2_O_3_/HfN metasurfaces	0.45 to 0.7	–	1	9.92 (660 nm)	2.58 × 10^12^ (660 nm)	729/146 ms (660 nm)	Enhanced light-matter interaction via resonance coupling	Single device	[174]
	Gr/Si Nanoholes	0.63	Schottky junction	1	2720 (635 nm)	1.25 × 10^11^ (635 nm)	6.2/8.6 µs (635 nm)	Efficient light trapping and transport	Single device	[173]
	Gr/GaAs nanocone array	0.85	Schottky junction	0	1.73 × 10^–3^ (850 nm)	1.83 × 10^11^ (850 nm)	72/122 μs (850 nm)	Efficient capture of incident photons	Single device	[143]
	Gr/Te-Si	0.27	Schottky junction	1	100 (1550 nm) 16.3 (2700 nm)	5.6 × 10^9^ (1550 nm) 0.93 × 10^9^ (2700 nm)	1.2/0.9 ms (1550 nm)	High photogain detectors working in the SWIR region at room temperature	Single device	[182]

In ideal scenarios, the band alignment of a heterojunction is determined by the work functions, electron affinities, and bandgaps of the constituent materials. However, in practical devices, interfaces inevitably host a high density of defect states, dangling bonds, and chemical hybridization, which give rise to Fermi-level pinning and band reconstruction, causing substantial deviations of the actual barrier height and band bending from ideal theoretical predictions (Table 3). To address these challenges, interface engineering implemented by introducing ultrathin passivation layers, tunneling layers, or functional interlayers (e.g., AlOₓ, h-BN, and SiO_2_) can effectively reduce the interface state density and alleviate Fermi-level pinning, thereby restoring or precisely tuning the intended band alignment. Simultaneously, interface engineering suppresses interfacial recombination and leakage currents, leading to a pronounced reduction in noise without significantly compromising photocarrier injection efficiency, and thus enables an overall enhancement in specific detectivity. This advantage is particularly pronounced in infrared and low-light photodetection, where device performance is often limited by noise. It should be noted that the effectiveness of interface engineering is highly sensitive to the thickness of the interfacial layer. Excessively thick layers introduce additional tunneling barriers that hinder carrier transport and degrade response speed, whereas overly thin layers fail to provide sufficient defect passivation, necessitating careful optimization to balance interface quality and transport efficiency.

**Table 3:** Advantages and disadvantages of different modulation mechanisms

Modulation Strategy	Advantages	Challenges
Band engineering	• High photocarrier collection efficiency • Overcomes intrinsic bandgap limitations • Enables multispectral, broadband, and tunneling-assisted sub-bandgap response	• Ideal band alignment can be strongly affected by interface defects, dangling bonds, and chemical hybridization • Actual barrier height and band bending can deviate significantly from theory
Interface engineering	• Suppresses interfacial recombination and leakage currents • Lower noise and increase detectivity	• Thickness optimization is critical • Some impact on the response speed
Electrical coupling	Dynamic control of photoresponse polarity, dynamic range, and spectral selectivity	• Requires additional electrodes and gate design • Increased device complexity and potential power consumption
Geometric/structural design	Programmable spatial control of photoresponse and carrier distribution	• High fabrication complexity • Challenges in device uniformity and large-area scalability

However, interface engineering alone is often insufficient to simultaneously achieve low dark current, high carrier separation efficiency, and fast response speed. Carrier separation efficiency is primarily governed by the spatial distribution of energy bands, while interface defects and barrier modulation critically affect carrier extraction efficiency [99]. Therefore, the synergistic optimization of interface engineering and band engineering is a key strategy for enhancing the overall optoelectronic performance of heterojunction devices. Such synergistic optimization relies on precise control of band alignment, carrier dynamics, and interface quality through rational material selection, defect engineering, and interface passivation [106]. For example, in type-II heterojunctions, the introduction of passivation layers can further suppress interfacial recombination and enhance the built-in electric field, thereby synergistically improving response speed and detectivity [108]. In Schottky-type structures, the insertion of an ultrathin interfacial layer introduces an additional tunneling barrier at the heterojunction interface, requiring carrier injection to proceed via tunneling [154, 160]. Consequently, even an interfacial layer with nanometer-scale thickness can significantly suppress thermionic-emission-dominated carrier transport under dark conditions.

Beyond static band design, its capability for functional reconfigurability and dynamic tunability remains inherently limited. In contrast, electric-field coupling takes advantage of the atomic-scale thickness and strong electrostatic modulation of two-dimensional materials, enabling dynamic tuning of the Fermi level, Schottky barrier height, and depletion width through gate biasing or external electrical stimuli. As a result, a single device can be reversibly switched among multiple operation modes, allowing electrical control over response polarity, dynamic range, and spectral selectivity, and even the integration of basic logic or synaptic-like functionalities. This transition from static high-performance operation toward dynamically programmable responses is closely aligned with the development of intelligent sensing, reconfigurable optoelectronic systems, and multifunctional integrated devices.

Furthermore, structural modulation introduces 3D micro-/nanostructured designs to generate spatially non-uniform strain fields, curvature, or geometric confinement in 2D materials or heterostructures, thereby enabling localized band modulation and position-dependent photocarrier distributions. This approach breaks the design constraints of conventional planar devices and allows programmable control of spectral response, photocurrent distribution, and functional behavior in the spatial domain, making it particularly attractive for multidimensional information acquisition and bio-inspired sensing applications. Nevertheless, challenges associated with fabrication complexity, device uniformity, and large-area scalability remain, underscoring the need for integration with mature microfabrication techniques and scalable manufacturing processes. Overall, the synergistic integration of these strategies is expected to be a key pathway for advancing 2D/3D heterojunction photodetectors from single-parameter optimization toward highly integrated, intelligent, and system-level applications.

## Application Based on 2D/3D PDs

### Imaging

Imaging is one of the core objectives in the research and application of photodetectors. Si integrated circuit technology has undergone decades of development, achieving a highly mature and reliable level that supports large-scale manufacturing and ultra-large-scale integration. In the field of photodetectors, meeting the requirements of device miniaturization, low-power consumption, and seamless integration with existing electronic components makes integration particularly important. Therefore, Si-based photodetectors, or other novel photodetectors compatible with CMOS processes, are regarded as primary candidates [56, 194–196]. By leveraging mature CMOS technology, not only can manufacturing costs be significantly reduced and device uniformity improved, but the monolithic integration of photodetectors with signal processing, data storage, and communication units can also be achieved, thereby enabling higher functional density and superior system performance. Such compatibility is of critical importance for advancing the development of next-generation on-chip optoelectronic integrated circuits.

**Fig. 11:**
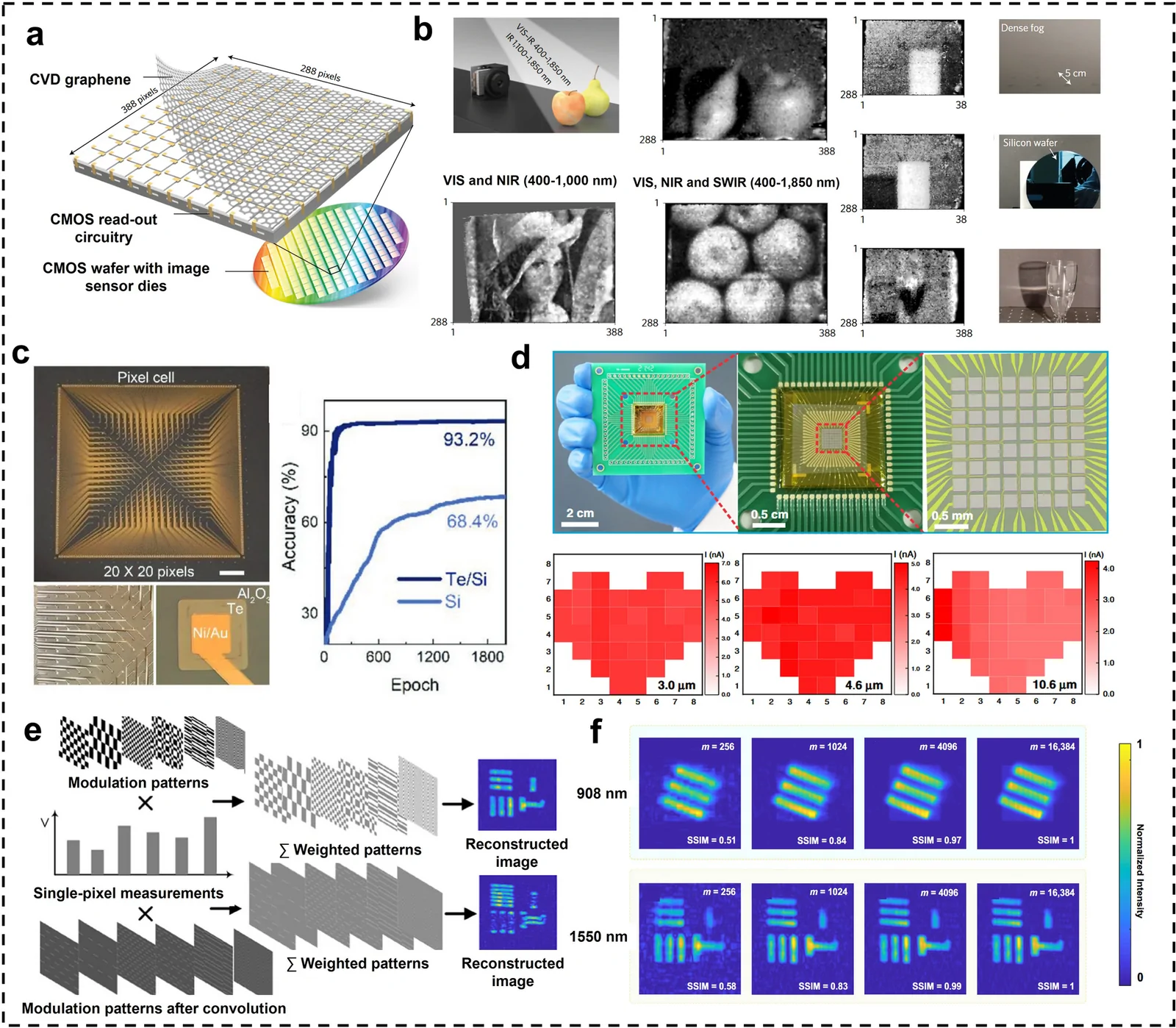
Applications of 2D/3D heterojunctions in imaging. **a** Computer-rendered impression of the CVD graphene transfer process on a single die (real dimensions 15.1 mm height, 14.3 mm width) containing an image sensor readout circuit that consists of 388 × 288 pixels. Reprinted with permission from Ref. [83]. Copyright (2017), Springer Nature. **b** Gr/CQD-based image sensor and digital camera system. Reprinted with permission from Ref. [201]. Copyright (2017), Springer Nature**. c** Optical photograph of imaging array (left). The right panel compares the recognition rates of Te/Si and Si arrays in image recognition. Reprinted with permission from Ref. [155]. Copyright (2023), John Wiley and Sons**. d** Photographs of an 8 × 8 1Tʹ-MoTe_2_/Si Schottky junction device array. The device area of each pixel cell is 200 × 200 μm^2^. Reprinted with permission from Ref. [62]. Copyright (2023), Springer Nature.** e** A schematic diagram of algorithm, calculating the inversion to obtain the image of the target object. **f** Restoration results of two objects under 908 and 1550 nm irradiation, respectively. Reprinted with permission from Ref. [202]. Copyright (2024), John Wiley and Sons

Compared with conventional photoactive materials, emerging 2D materials exhibit superior performance in terms of compatibility, scalability, spectral detection range, and sensitivity, thereby demonstrating tremendous potential for imaging applications [197–200]. In contrast to the integration challenges faced by non-silicon photoactive materials with silicon integrated circuits, the advantages exhibited by graphene and related 2D materials underscore the critical importance of incorporating 2D materials into next-generation microelectronic devices, sensor arrays, and low-power integrated photonic systems. In 2017, Goossens et al. achieved monolithic integration of graphene with CMOS integrated circuits, realizing a digital camera (388 × 288 array) sensitive to ultraviolet, visible, and infrared light (300–2000 nm) [83]. This proof-of-concept monolithic CMOS image sensor represents a milestone for the development of low-cost, high-resolution broadband and hyperspectral imaging systems (Fig. 11a, b). However, the standard growth methods for many low-dimensional materials, such as high-temperature CVD and epitaxial growth, are incompatible with the temperature and material constraints of CMOS processes. Therefore, exploiting the excellent optoelectronic properties of these materials in integrated systems remains a significant challenge. Considering the high stability, efficient hole transport capability, superior optoelectronic properties, and simple fabrication process of Te, Li et al. achieved monolithic integration of large-area Te-based optoelectronic functional units through magnetron sputtering. They demonstrated a Te/Si heterojunction-based imaging array (20 × 20 pixels) [201]. Owing to the high contrast obtained from the Te/Si array compared with the Si array, applying electronic images to artificial neural networks (ANNs) for mimicking artificial visual systems significantly improved the efficiency and accuracy of subsequent processing tasks (Fig. 11c). ANNs are computational models inspired by biological neural systems and are used for tasks such as pattern recognition, classification, and prediction.2D materials present distinct advantages for infrared photodetectors, including tunable bandgaps, high sensitivity, and room-temperature operation. In contrast, state-of-the-art infrared detectors, based on narrow-bandgap semiconductors such as In_1−x_Ga_x_As, InSb, and Hg_1−x_Cd_x_Te, require costly growth techniques (e.g., Molecular Beam Epitaxy (MBE) and Liquid Phase Epitaxy (LPE)) and low-temperature cooling to suppress dark current. These requirements increase power consumption, system complexity, and overall cost, while their poor CMOS compatibility limits practical integration. In 2023, Wu et al. fabricated large-area uniform 2D MoTe_2_ using a thermally assisted tellurization method and assembled an 8 × 8 focal plane array devices (MoTe_2_/Si), demonstrating impressive MIR imaging capability without the need for low-temperature cooling (Fig. 11d) [62]. This work provides a feasible approach for the development of high-sensitivity 2D photodetectors for room-temperature MIR photodetection and imaging applications. Furthermore, in 2024, Shi et al. proposed an advanced computational imaging system that employs a MoTe_2_/Si self-powered photodetector combined with a single-pixel imaging (SPI) algorithm based on Hadamard spectra [202]. This system achieved high-resolution (128 pixels) short-wave infrared imaging and high-quality edge reconstruction under a low sampling rate of 25% (Fig. 11e, f).

In recent years, 2D/3D heterojunction photodetectors have achieved remarkable progress in single-pixel performance and the demonstration of small-scale arrays; however, several critical limitations remain for their application in imaging. First, insufficient device uniformity and scalability continue to hinder the development of high-resolution focal plane arrays (FPAs), resulting in a significant gap compared with the megapixel-level commercial InGaAs or HgCdTe FPAs. Second, inadequate suppression of noise and dark current leads to a D* that is still inferior to that of conventional narrow-bandgap semiconductors in the mid-infrared and long-wavelength infrared, with strong dependence on wavelength and illumination condition. Overall, while 2D/3D heterojunction photodetectors exhibit unique potential for imaging in the SWIR/MIR regimes, their practical deployment in large-scale imaging systems still requires breakthroughs in material uniformity, noise engineering, optical coupling, and system-level integration.

### Intelligent Machine Vision

Machine vision is a critical component of artificial intelligence and automation systems, with broad applications in autonomous driving, security monitoring, industrial inspection, and human–computer interaction [203–205]. Conventional vision systems are typically composed of three modules: a photodetector for imaging, an analog-to-digital converter for data conversion, and a processor for executing intelligent algorithms. However, with the rapid emergence of application scenarios such as autonomous vehicles and embedded artificial intelligence (AI), which demand higher levels of real-time performance, energy efficiency, and intelligence, the traditional “sense-store-compute” decoupled architecture is increasingly constrained by several bottlenecks [206–208]. On the one hand, frequent data transfer between the photodetector, memory units, and processor incurs substantial energy consumption and latency, making it difficult to meet the stringent requirements of edge devices for low power and low delay. On the other hand, conventional vision systems often struggle to process complex and dynamic scenes, where high data redundancy, significant background noise, and difficulties in target feature extraction result in reduced recognition accuracy and slower response in subsequent algorithms. As an emerging paradigm, edge brain-inspired image sensors with built-in neural networks and in-sensor processing architectures offer a promising solution to these challenges [208–222].

**Fig. 12:**
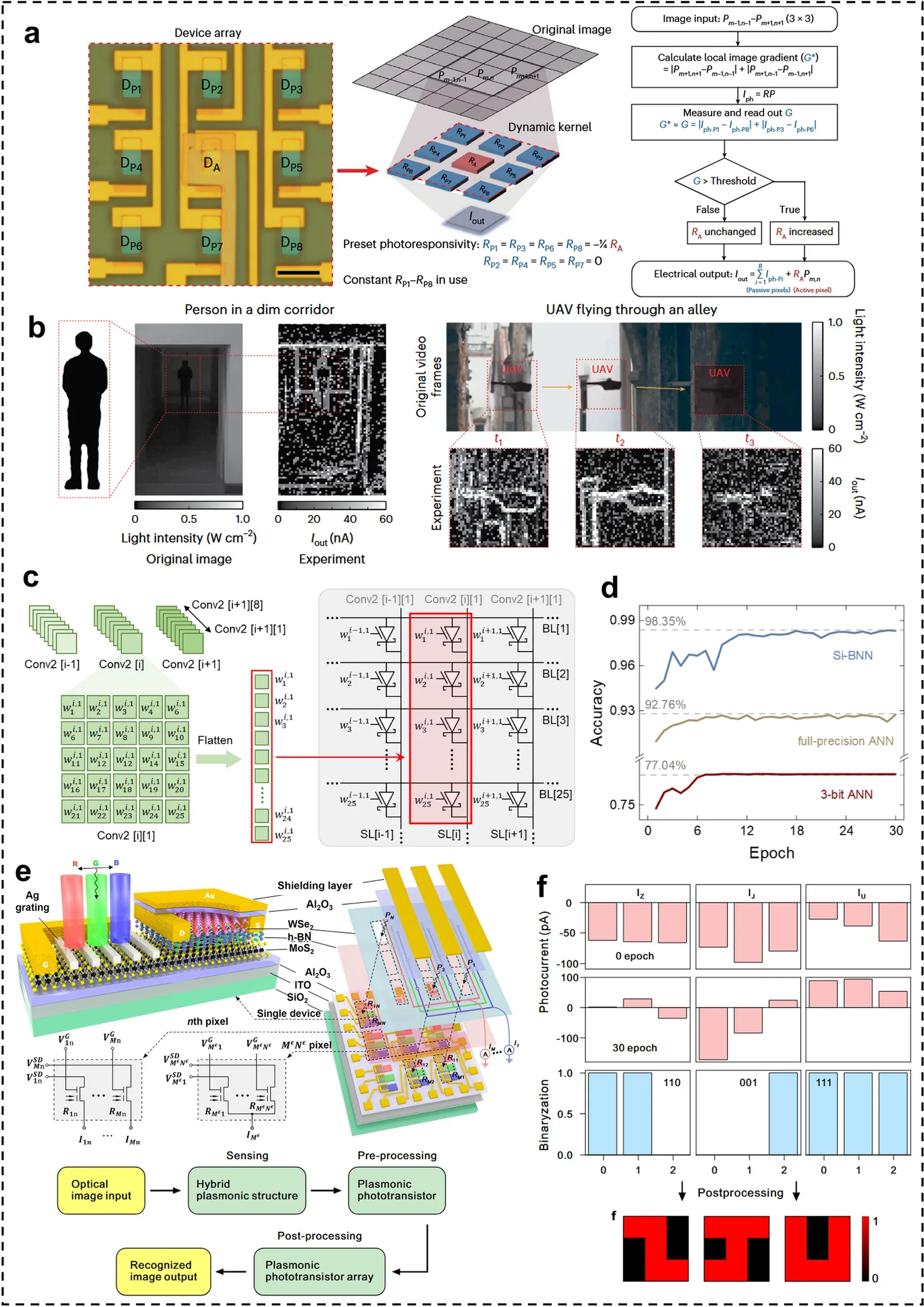
Applications of 2D/3D heterojunctions in intelligent machine vision. **a** Optical image and an algorithm flow chart for extracting edge features based on the graphene/Ge heterostructure. The symbols DA and DP1-DP8 denote active device and passive devices, respectively. **b** Experimental results of in-sensor dynamic computing demonstrate clear edge distinction in the processed image. **a, b** Reprinted with permission from Ref. [86]. Copyright (2024), Springer Nature.** c** Mapping schematic of a 2D kernel from the second convolution layer on the quasi-binary MAC engine. **d** Training histories of the SI-BNN, 3-bit precision ANN, and full-precision ANN on the MNIST dataset. **c, d** Reprinted with permission from Ref. [73]. Copyright (2024), Springer Nature.** e** Schematic of a 2D phototransistor array. The bottom panel shows illustration of an AVPRM based on the phototransistor array for image preprocessing and an ANN for image recognition. **f** Reconstructed letters after post-processing. **e, f** Reprinted with permission from Ref. [78]. Copyright (2025), American Chemical Society

In 2024, Yang proposed a novel intelligent machine vision approach based on in-sensor dynamic computing, which leverages multi-terminal graphene/Ge heterostructure device arrays to achieve dynamic photoresponse modulation and spatially correlated processing at the sensor level [86]. Unlike conventional vision systems that require image data to be transmitted to back-end processors for edge extraction, this method constructs dynamic convolutional kernels through the coupling of active and passive devices. As a result, the photoresponse of the central device adaptively adjusts to the intensity differences of surrounding pixels, thereby enabling selective amplification and real-time extraction of edge features directly in the analog domain (Fig. 12a). The underlying mechanism relies on the photovoltaic effect and gate-tunable Schottky barriers within the device, where electrical feedback modulates the photoresponse characteristics to realize in-sensor fusion of sensing and computation. This improves the accuracy, speed, and energy efficiency of weak target detection under low-illumination and low-contrast conditions, offering a hardware-level real-time processing pathway for intelligent vision systems in complex environments such as autonomous driving and security monitoring (Fig. 12b).

Compared with Yang’s demonstration of dynamic edge extraction within the sensor, Chen et al. realized a complete in-sensor binary neural network by constructing programmable graphene/silicon Schottky diodes [73]. By applying gate voltages, the photoresponse was discretized into {+ 1, − 1} weights and mapped into a crossbar array, thereby enabling quasi-binary MAC operations that simultaneously executed Conv1, Conv2, FC1, and FC2 layers (Fig. 12c). This architecture boosted the recognition accuracy on the MNIST dataset from 77% to 98.35% (Fig. 12d). Stronger image understanding capabilities and system-level intelligence have attracted considerable interest from researchers. Zhang et al. proposed an artificial visual perception and recognition module (AVPRM) based on a plasmon-enhanced 2D material phototransistor array (Fig. 12e) [78]. The device exploits Ag nanograting/MoS_2_ plasmonic structures to induce localized surface plasmon resonance, generating hot electrons that enhance and modulate photoresponse under gate and thermoelectric control. A built-in floating gate transfers processed photocurrent to a WSe_2_ channel, mimicking synaptic weight modulation. By programming pixel photoresponsivity via source-drain bias, the array performs on-chip preprocessing (contrast enhancement, noise suppression) and, combined with a perceptron neural network, enables image recognition (Fig. 12f). It exhibits an ultrawide dynamic range (180 dB), ultrafast response (500 ns), and ultralow energy consumption (2.4 × 10^–17^ J pulse^−1^).

### Logical Operations

**Fig. 13:**
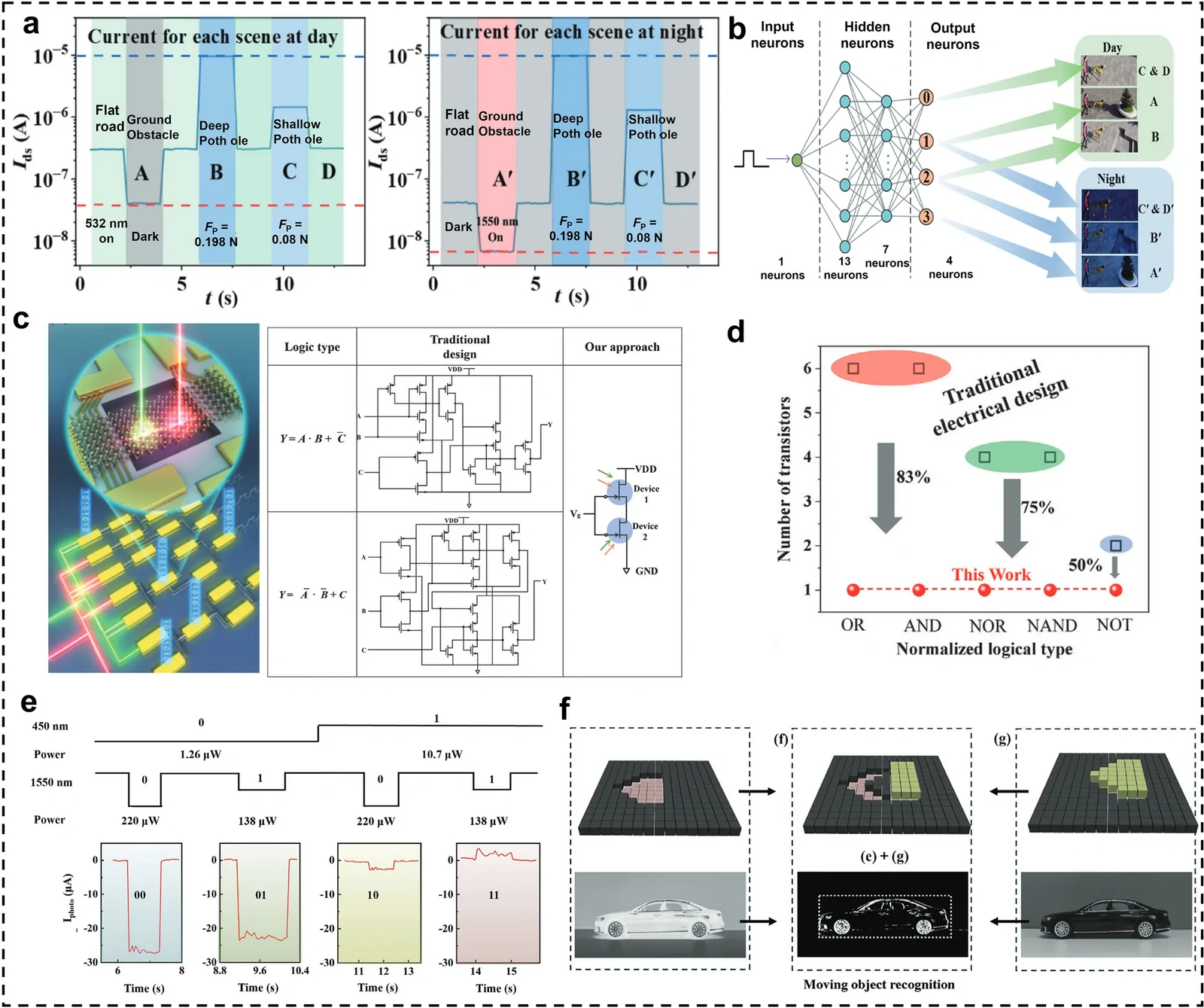
Applications of 2D/3D heterojunctions in logical operations. **a** Output currents of electronic guide dogs (EGDs) under various scenarios during the day (left) and night (right). **b** Schematics of the process and parameters for constructing the DNN structure. **a, b** Reprinted with permission from Ref. [227]. Copyright (2025), John Wiley and Sons.** c** Schematic diagram of an all-optical logical circuit. **d** Comparison of the circuit diagrams (left) and the transistor number of (right) complex logical operations between the traditional design and the all-optical device. **c, d** Reprinted with permission from Ref. [71]. Copyright (2023), Springer Nature.** e** By selecting two power levels of 450 and 1550 nm, four types of logic signal outputs can be achieved. **f** Image is constructed by summing the results of two output current matrices, which facilitates the identification of object motion trajectories. **e, f** Reprinted with permission from Ref. [69]. Copyright (2024), John Wiley and Sons

In electronic circuits, complex computational tasks are accomplished by interconnecting various logic units. Although electrical logic operations have undergone extensive development, the continuous scaling of semiconductor process nodes has introduced challenges such as increased power consumption, reduced stability, and significant thermal effects during electrical signal processing [223, 224]. With the surge in demand for AI and high-performance computing, the parallel computing capabilities of traditional CPUs/GPUs are increasingly struggling to meet the requirements of complex scenarios. In contrast, photons offer distinct advantages over electrons, including lower power consumption and minimal thermal limitations. Furthermore, photonic devices inherently support parallelism and high bandwidth, holding great promise for delivering substantial computational power [225, 226]. Han et al. monolithically integrated a MoS_2_/Ge heterojunction phototransistor with a piezoresistor, exploiting its visible–infrared dual-band bipolar photoresponse (+ 182 A W^−1^ at 532 nm and − 37 A W^−1^ at 1550 nm) together with electro-optical co-modulation to implement multimodal-perception and scene-classification logic on a single device (Fig. 13a) [227]. By encoding optical/pressure stimuli as input variables, a shallow neural network was constructed that achieved ~ 100% classification accuracy across seven typical road conditions after only 48 training epochs (Fig. 13b).

The conditional switching between daytime passive vision and nighttime active infrared operation functions as an on-device conditional logic gate, markedly reducing power consumption. In 2024, You et al. further exploited the wavelength- and power-reconfigurable bipolar photoresponse of the same MoS_2_/Ge heterojunction phototransistor, upgrading the material platform into a complete, reconfigurable, and array-compatible all-optical logic architecture. Leveraging the device’s positive photoresponse under VIS light and negative photoresponse under NIR light, they realized all five fundamental all-optical logic gates (AND, OR, NOT, NAND, and NOR) on a single transistor. By precisely tuning the wavelength and power of the input optical signals, distinct logic functions can be dynamically reconfigured. Moreover, cascading two such devices enable the execution of complex logical operations such as Y = A·B + C̄ (Fig. 13c) [71]. Compared with conventional electronic logic circuits, this approach reduces the required transistor count by 50%–94% for equivalent functionalities, offering significant advantages in integration density, power efficiency, and operational fidelity (Fig. 13d). In 2025, Yang et al. advanced the bipolar response paradigm into a continuously, symmetrically, and ultra-broadly tunable power-modulation regime. By fixing a 450 nm signal beam and a 1550 nm modulation beam, they showed that sweeping the modulation power enables the responsivity to cross zero linearly from − 3376 to + 3584 A W^−1^, thereby realizing real-time, continuous, and reconfigurable logic state switching (Fig. 13e). Compared with MoS_2_/Ge heterojunction phototransistors (positive and negative responsivities differ by nearly two orders of magnitude and usable window is constrained by the smaller amplitude) the CdS/graphene/Ge photodetector exhibits almost symmetric PPC and NPC with a tuning range enlarged by ~ 3 orders of magnitude [69]. This wide, symmetric dynamic range allows the population of larger computational matrices and the allocation of more threshold levels, directly enhancing logic depth and recognition resolution (Fig. 13f).

### Optoelectronic Integrated Systems

With the rapid advancement of AI and big data, the computational power demands of chips continue to increase, necessitating new data transmission material platforms with high bandwidth, low-power consumption, compact footprint, and low cost. Conventional chips use metallic interconnects for data transmission, but as integration density improves, these interconnects face significant challenges such as signal delay and power consumption, which hinder further improvements in computational performance. On-chip optical interconnect technology replaces electrical wires with optical waveguides. Thanks to their low latency and minimal power dissipation, optical links can achieve much higher data transmission rates [228, 229]. Moreover, since optical signal propagation is not affected by electrical resistance, they allow longer interconnect distances. On-chip optical interconnects are considered a key pathway to enhancing single-chip computational performance in the future [71, 230–234]. This progress further drives the development of next-generation high-performance optoelectronic integrated systems (OEIS) based on monolithic integration of nanophotonics and nanoelectronics. Such systems aim to realize multifunctional intelligent micro-systems that integrate sensing, transmission, processing, and computing on a single chip [230]. The realization of OEIS has been hindered by lattice mismatch between different materials required for different device components. Although monolithic OEIS have been demonstrated using standard Si CMOS foundry processes [230, 235], the performance of the fabricated photodetectors remains limited and struggles to compete with those based on compound semiconductors.

**Fig. 14:**
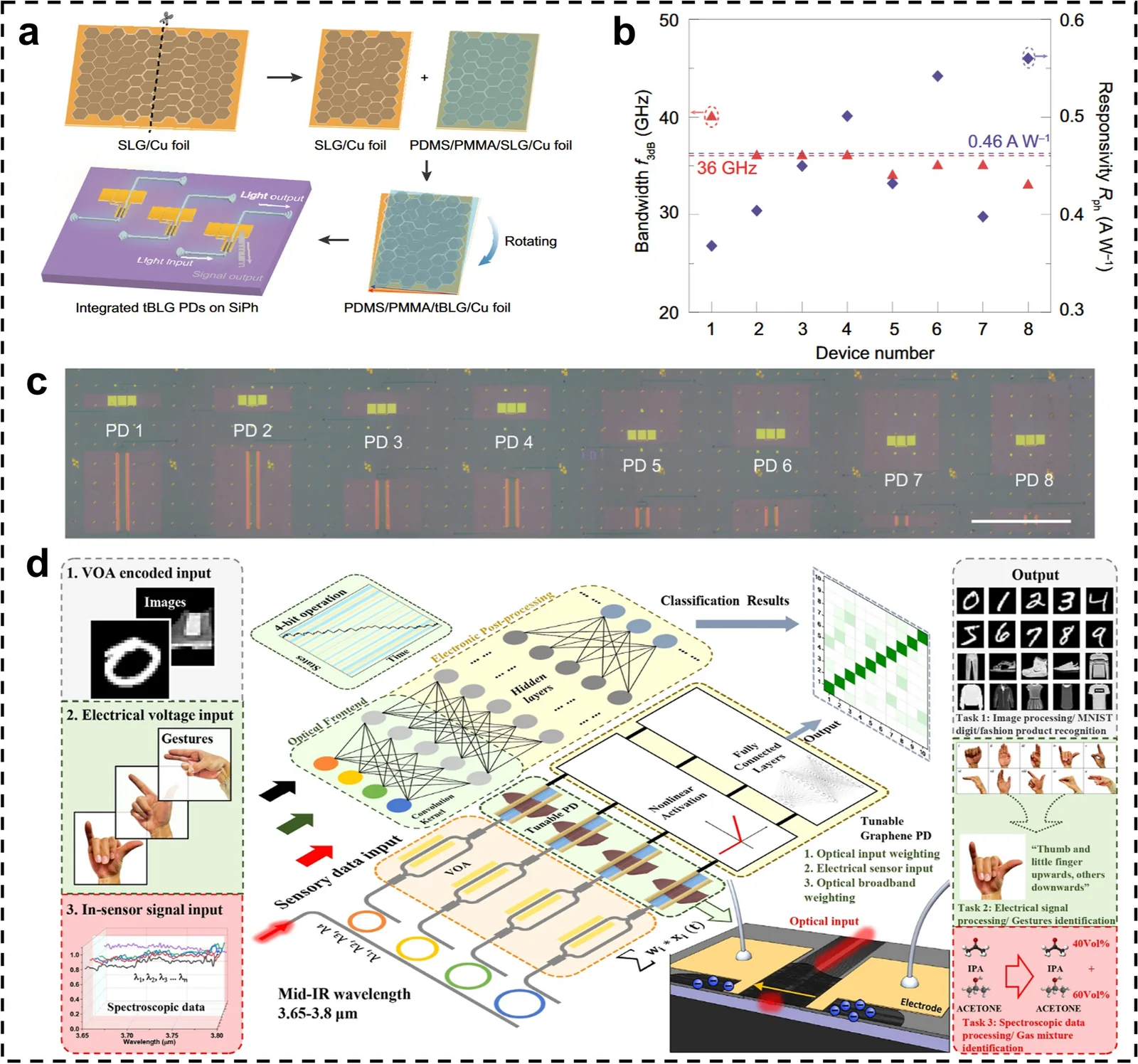
Applications of 2D/3D heterojunctions in optoelectronic integrated systems. **a** Schematic illustration of the large-scale transfer of tBLG and fabrication of tBLG photodetectors. **b** Statistical histogram of photoresponsivity and 3 dB bandwidth. **c** Optical image of tBLG photodetectors array. **a–c** Reprinted with permission from Ref. [242]. Copyright (2024), Springer Nature. **d** Schematics of the proposed waveguide-integrated MIR optoelectronic processing unit. MIR light is coupled into the multiple waveguide channels, each is encoded with sensory data signal. The optical signal is then picked up by the graphene photodetector, whose weight is set by adjusting the bias voltage. The multiplied signals are then summed and sent to electrical post-processing for classification tasks. Reprinted with permission from Ref. [243]. Copyright (2024), American Chemical Society

Owing to their intrinsic properties, 2D materials are emerging as a significant development direction in the field of silicon photonics, promising to bring multiple advantages to on-chip optical interconnection systems, including high-performance, low-power consumption, broad bandwidth, compact structure, and process compatibility. For instance, single-layer graphene (SLG) with high carrier mobility has been integrated with silicon photonic, demonstrating high bandwidth [236, 237]. A key challenge limiting the adoption of SLG in optical receivers is its intrinsically low photoresponsivity compared with conventional photodetectors. Considerable efforts have been devoted to enhancing the photoresponsivity of graphene-Si photonic photodetectors through integrated external absorption enhancement structures, such as microring resonators [238], plasmonic-assisted architectures [239, 240], photonic crystal waveguides [241] and light-absorbing layers. Wu et al. integrated twisted bilayer graphene (tBLG) into Si photonics to enhance optical absorption in the photodetector channel (Fig. 14a, c) [242]. They achieved a high responsivity of up to 0.65 A W^−1^ at the communication wavelength of 1550 nm, a 3 dB bandwidth exceeding 65 GHz, and supported a data rate of 50 Gbit s^−1^ (Fig. 14b).

The high responsivity is attributed to the enhanced optical absorption resulting from van Hove singularities in tBLG, as well as the strong electric field at the electrode/graphene interface within the device structure. Liu et al. further proposed an on-chip in-sensor computing system based on a MIR silicon waveguide platform [243]. The waveguide-integrated graphene detector is extended to the mid-infrared (3.65–3.8 μm), and its electrically tuned responsivity is innovatively utilized to realize analog multiply–add operations with 4-bit precision. The optical signal is transmitted through a waveguide, encoded by a variable optical attenuator, and then multiplied by a graphene detector with “photoelectric-weighting”. The output current is summed and sent to the subsequent neural network for classification. Successful demonstration of photoelectric intra-sensing computation in image preprocessing, gesture recognition (with the accuracies of 72.43%), and gas spectral analysis (with the accuracies of 87.14%), and successful integration of mid-infrared photon sensing and neural network computation on the same chip (Fig. 14d).

### Position-Densitive Detector

High-speed motion capture and multi-target trajectory tracking are crucial in numerous application fields. However, conventional high-speed imaging technologies whether based on CCD or CMOS image sensors inevitably involve a trade-off between image resolution and frame rate [244, 245]. In contrast, position-densitive detectors (PSDs) which operate based on the lateral photovoltaic effect, offer a promising alternative for single-spot tracking. They not only achieve high positioning accuracy but also provide sub-micrometer spatial resolution. The band structure of materials and carrier mobility are crucial factors influencing the lateral diffusion mechanism of photogenerated carriers. Owing to the high carrier mobility of 2D materials, PSDs based on these materials exhibit high positional sensitivity and ultrafast photoresponse [244, 246–255]. Their compatibility with Si technology enables diverse optoelectronic applications.

**Fig. 15:**
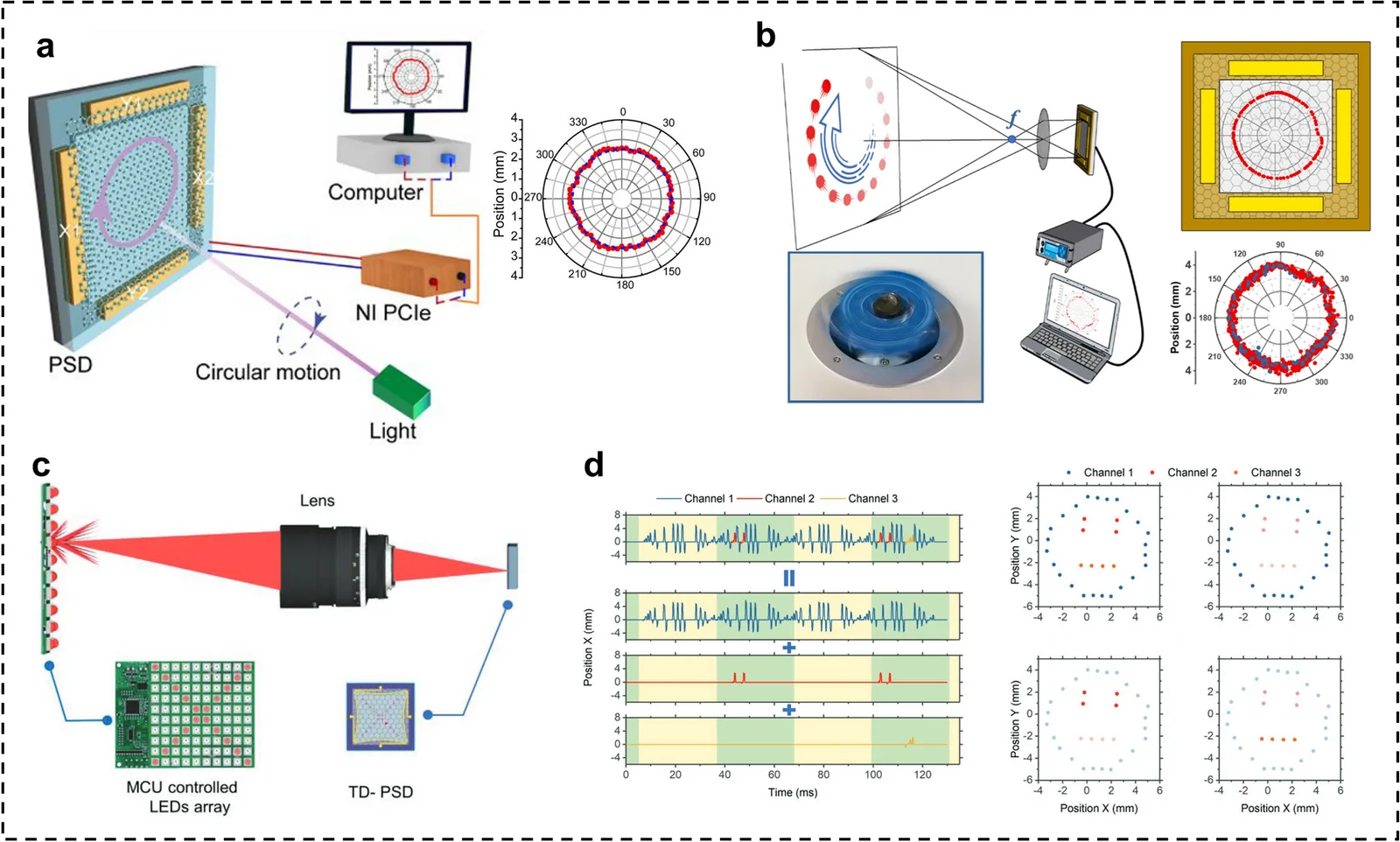
Applications of 2D/3D heterojunctions in PSD. **a** Schematic of the light trajectory measurement (left) and the light trajectory measurement result acquired from the PSD (right). Reprinted with permission from Ref. [252]. Copyright (2022), American Chemical Society.** b** High-speed trajectory tracking using graphene-Ge PSD. Reprinted with permission from Ref. [247]. Copyright (2019), American Chemical Society.** c** Setup of the system. The MCU-controlled LED array provides high-frequency switching multi-target light sources. Then, the images are projected to the graphene/Si position-sensitive detector through a lens. **d** Multichannel tracking signal and the corresponding separated subpattern signals extracted by the system. The right panel displays the reconstructed face patterns. Reprinted with permission from Ref. [244]. Copyright (2022), John Wiley and Sons

Chen et al. developed a solar-blind PSD based on a graphene/Ga_2_O_3_ heterojunction, which leverages multibeam interference to enhance absorption [252]. At a wavelength of 250 nm, the device achieved a responsivity of 48.5 mA W^−1^ and a sub-millisecond response time under zero bias. It also exhibited high-temperature stability (up to 200 °C) and enabled high-precision angular displacement and trajectory measurements (Fig. 15a). Furthermore, its potential in optical communication signal demodulation was demonstrated. Liu et al. extended the operational spectrum of PSDs to the NIR region by constructing a graphene/germanium heterojunction PSD [247]. This device exhibited nW weak-light detection capability and micrometer-scale spatial resolution, successfully enabling high-speed target trajectory tracking (> 100 km h^−1^) and sound frequency recovery, thereby validating its utility in precision measurement and high-speed infrared sensing (Fig. 15b). However, conventional PSDs are unable to distinguish multiple targets based on mixed carrier concentration distributions, highlighting the urgent need for a multi-target trajectory tracking system with high frame rates, superior resolution, and real-time data-processing capabilities. To address this, Liu et al. proposed a time-division PSD based on a graphene/Si heterojunction [244]. By exploiting the lateral diffusion of carriers and the high-speed photoresponse mechanism, the system achieved multi-target trajectory tracking and image reconstruction at frame rates of up to 62,000 fps, significantly outperforming conventional CCD/CMOS imaging systems (< 1000 fps). The system also demonstrated capabilities in multi-channel target recognition and ambient light interference suppression (Fig. 15c, d).

## Summary and Outlook

**Fig. 16:**
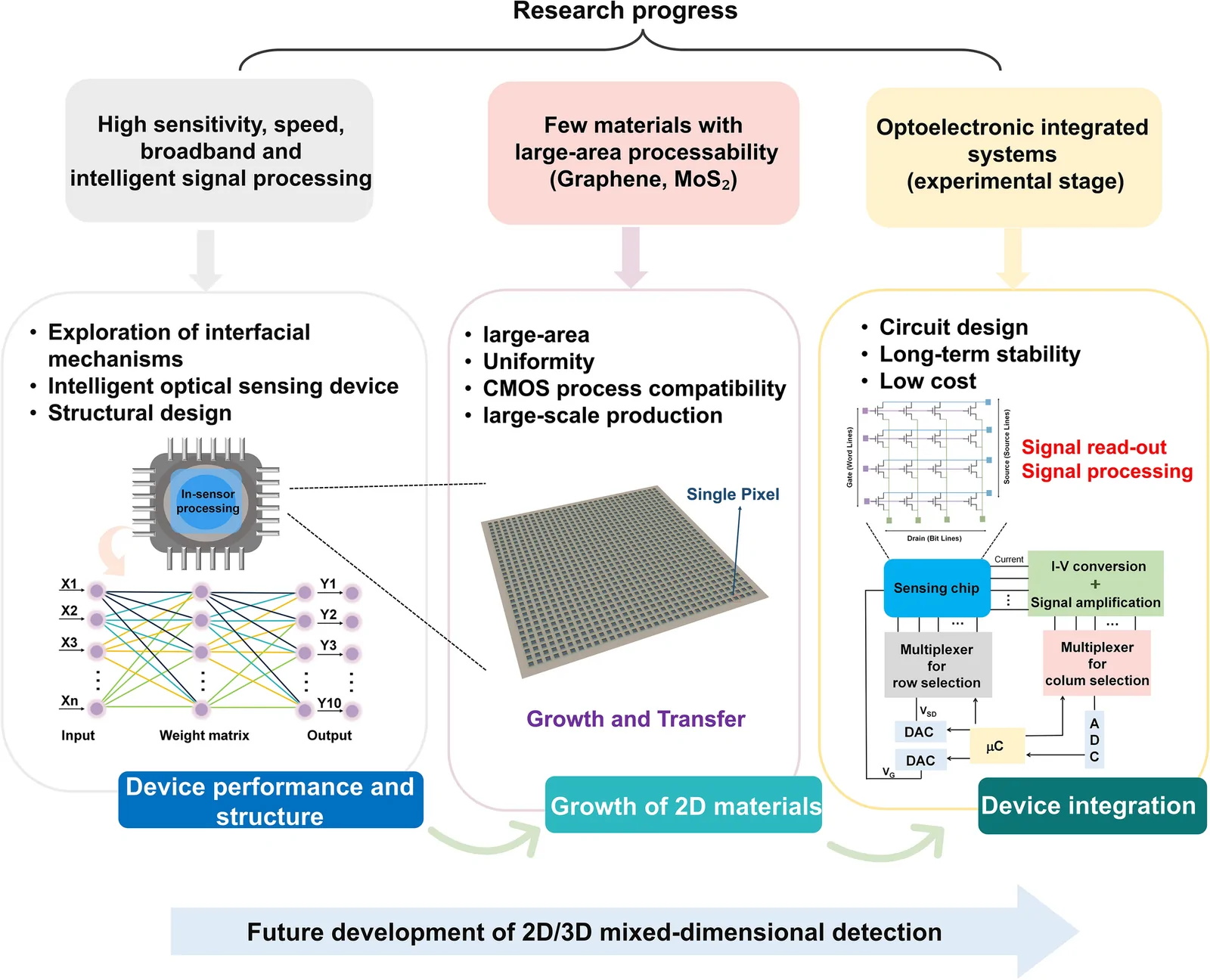
The research progress and future development trends

2D materials, with their atomic thickness, tunable band structures, and superior interfacial properties, offer unique advantages for achieving highly sensitive, broadband, and ultrafast photodetection. In contrast, 3D semiconductors possess mature fabrication processes and stable electrical and optical properties, laying a solid foundation for large-scale device applications. The heterogeneous integration of the two not only overcomes the limitations of conventional material systems in bandgap engineering and detection range but also opens up new opportunities for the development of multifunctional, low-power optoelectronic devices. Moreover, the direct coupling of high-performance photodetectors with circuits and computing modules paves the way for real-time signal processing, edge computing, and multimodal sensing. Such synergistic integration shortens the signal transmission pathway, reduces energy consumption, and enhances functional density, thereby establishing a robust foundation for the advancement of next-generation intelligent optoelectronic systems. However, from a commercialization perspective, although 2D/3D heterojunction-based optoelectronic devices have achieved rapid progress, several critical challenges remain to be addressed. This section analyzes the development and existing challenges of 2D/3D heterojunction optoelectronic devices from three perspectives: device performance, material synthesis, and device integration (Fig. 16).

### Device Performance and Structure

The applications of 2D/3D heterojunctions in photonic devices are rapidly expanding, offering significant potential for high sensitivity, broadband detection, and intelligent signal processing. Nevertheless, from the standpoint of device architecture and performance optimization, understanding of interfacial phenomena remains limited. Critical processes at the interface such as charge transfer dynamics and defect-state modulation play decisive roles in carrier transport, photoresponse efficiency, and device stability, yet they have not been fully elucidated. A deeper insight into these mechanisms will provide essential guidance for the rational design of high-performance and predictable optoelectronic systems.

At the same time, the convergence of AI and optoelectronic technologies has accelerated the development of intelligent sensing systems, imposing greater demands on optical sensors capable of information acquisition, real-time analysis, and autonomous decision-making. Accordingly, future research can prioritize: (1) In-depth investigation of interfacial physical mechanisms, clarifying how carrier dynamics, band structure modulation, and defect states influence device performance; (2) Development of optoelectronic devices with intelligent signal processing capabilities, enabling in situ data processing, feature extraction, and preliminary decision support at the sensing front end; (3) Design of novel architectures compatible with system integration, facilitating the translation from individual devices to modular, chip-level, and system-level implementations.

### Growth of 2D Materials

**Fig. 17:**
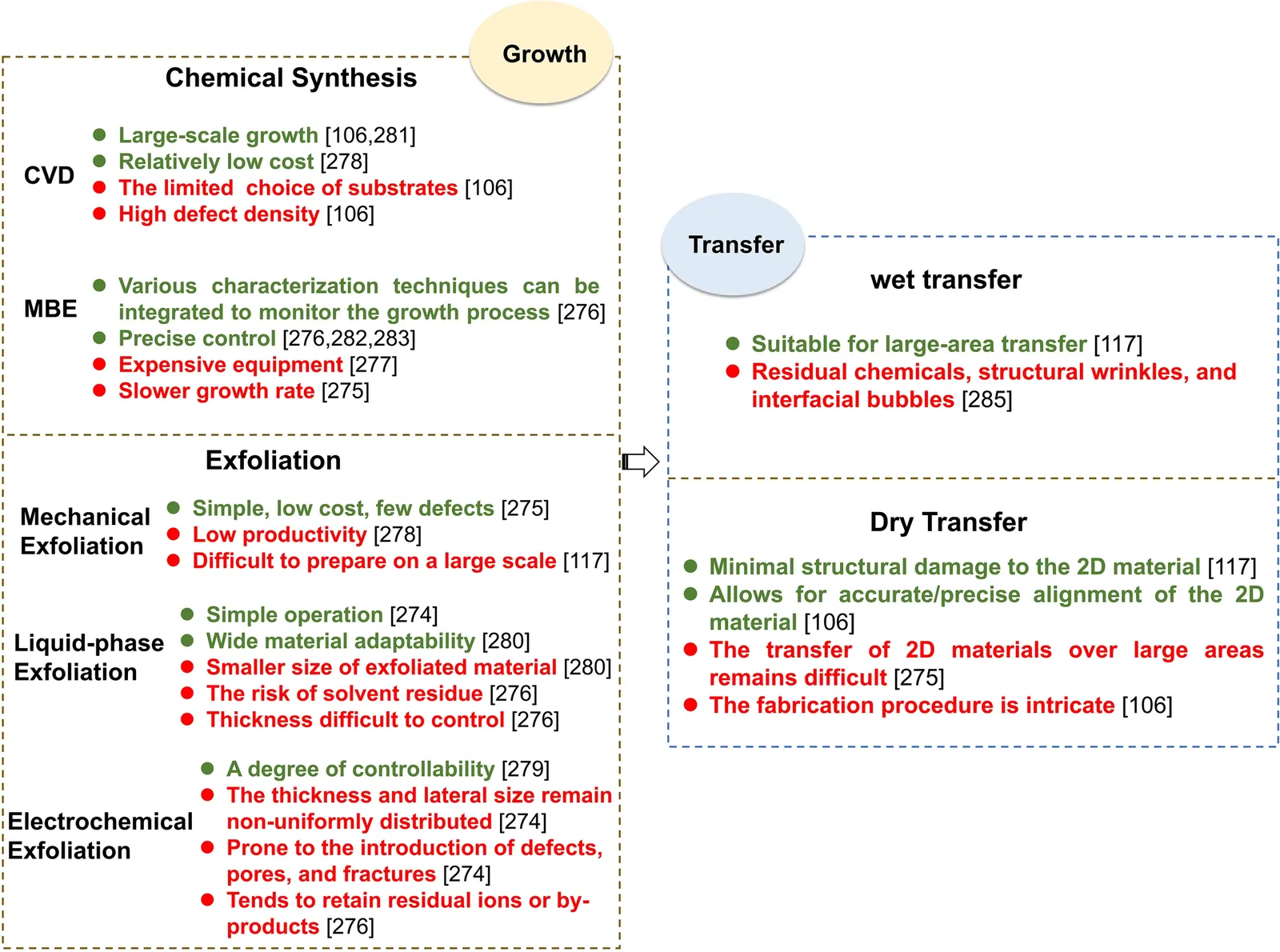
Growth and transfer strategies of 2D materials [106, 116, 267–276]

In the transition from laboratory research to practical applications, achieving large-area, high-quality, and controllable growth of 2D materials, optimizing interface engineering, and developing low-cost, scalable integration processes compatible with CMOS technology become critical requirements. Although 2D materials exhibit outstanding electrical, optical, and mechanical properties, commonly used synthesis methods still face challenges in terms of crystal quality, layer uniformity, and reproducibility, which hinder large-scale industrial production (Fig. 17) [52, 256–266]. Moreover, the growth temperature, chemical environment, and post-processing conditions of certain 2D materials are often incompatible with conventional silicon-based fabrication processes, posing significant challenges for their integration into existing semiconductor manufacturing platforms. At the same time, differences between 2 and 3D materials in terms of thermal expansion coefficients, interfacial energies, and chemical stability can lead to stress, dislocations, and impurity incorporation during deposition or transfer, thereby degrading interfacial quality and adversely affecting device performance.

Therefore, breakthroughs in key technologies are essential for achieving high-quality heterogeneous integration and industrial-scale deployment of 2D material-based devices, including low-temperature epitaxy, damage-free transfer, interface passivation, and encapsulation. Future research can further focus on: (1) Large-area, controllable, and low-defect 2D material fabrication techniques, including optimization of CVD, MBE, and liquid-phase epitaxy processes to meet industrial-scale production requirements; (2) Interface engineering strategies, such as surface modification, atomic-layer passivation, or functionalized interface materials, to improve carrier transport efficiency, reduce interface defect density, and enhance device stability; (3) Flexible-compatible and low-temperature integration processes, enabling 2D materials to be compatible with existing CMOS or other semiconductor platforms and broadening their potential application scenarios; (4) Scalable industrial processes, developing low-cost, highly reproducible, and standardized production workflows to support the transition from single-lab samples to chip-level and system-level devices.

### Device Integration

The direct coupling of high-performance photodetectors with circuit and computing modules opens new avenues for real-time signal processing, edge computing, and multimodal sensing. Such synergistic integration can shorten signal transmission paths, reduce power consumption, and enhance functional density, thereby laying a solid foundation for the development of next-generation intelligent optoelectronic systems [13, 277–279]. From the perspective of device integration, current transfer and alignment techniques for 2D materials still face uncertainties. Grain boundaries, thickness non-uniformity, and stress concentrations in 2D materials can lead to fluctuations in their electrical and optical properties. Due to the unique material characteristics and performance of 2D materials, conventional design approaches used for silicon-based circuits cannot be directly applied. In this context, the design of signal processing circuits, as well as associated packaging and testing technologies, remains a critical challenge.

Moreover, the development of high-resolution sensor chips inevitably increases crosstalk during the signal readout process. At the same time, the sensitivity of 2D materials such as MoS_2_ to ambient air can result in performance degradation, necessitating protective measures such as encapsulation to ensure long-term device stability. Building on this, future research can focus on: (1) Optimization of high-precision 2D material transfer and alignment techniques, minimizing the impact of grain boundaries, thickness non-uniformity, and stress concentrations on device performance; (2) Novel circuit design and packaging strategies, tailoring signal processing circuits to the specific properties of 2D materials in order to improve array uniformity and reduce noise; (3) Enhancement of environmental stability and durability, employing encapsulation, surface modification, or materials engineering approaches to improve device reliability under practical operating conditions; (4) Signal processing strategies for high-resolution arrays, combining hardware and software co-optimization to reduce crosstalk and achieve accurate multi-channel signal acquisition.

### Standardization and Performance Evaluation for Emerging and Intelligent Photodetectors

Despite the rapid progress of 2D/3D vdW heterojunction photodetectors, the lack of standardized characterization and testing protocols remains a critical challenge for fair performance evaluation and benchmarking. Key metrics such as responsivity, detectivity, and noise are often measured under different illumination conditions, device area definitions, bandwidths, and noise assumptions, making direct comparison across reports difficult and sometimes misleading. Recent guideline studies on emerging photodetector technologies have emphasized the importance of unified measurement conditions and rigorous noise characterization to ensure reliable and comparable performance metrics [95].

Beyond conventional photodetectors, this challenge becomes even more pronounced for emerging intelligent photodetectors, such as neuromorphic and synaptic optoelectronic devices. In these systems, traditional figures of merit alone are insufficient, as device performance is also governed by functionality-oriented parameters, including synaptic weight modulation, temporal response dynamics, learning accuracy, energy efficiency, and operational endurance. Establishing application-oriented and standardized evaluation methodologies that jointly consider optoelectronic performance and neuromorphic functionality will be crucial for enabling meaningful comparisons and accelerating the practical deployment of intelligent sensing and neuromorphic vision systems based on 2D/3D heterostructures.
